# Cervical lymph nodes and ovarian teratomas as germinal centres in NMDA receptor-antibody encephalitis

**DOI:** 10.1093/brain/awac088

**Published:** 2022-03-24

**Authors:** Adam Al-Diwani, Jakob Theorell, Valentina Damato, Joshua Bull, Nicholas McGlashan, Edward Green, Anne Kathrin Kienzler, Ruby Harrison, Tasneem Hassanali, Leticia Campo, Molly Browne, Alistair Easton, Hooman Soleymani majd, Keiko Tenaka, Raffaele Iorio, Russell C Dale, Paul Harrison, John Geddes, Digby Quested, David Sharp, Soon Tae Lee, David W Nauen, Mateusz Makuch, Belinda Lennox, Darren Fowler, Fintan Sheerin, Patrick Waters, M Isabel Leite, Adam E Handel, Sarosh R Irani

**Affiliations:** Oxford Autoimmune Neurology Group, Nuffield Department of Clinical Neurosciences, University of Oxford, Oxford, UK; University Department of Psychiatry, University of Oxford, Oxford, UK; Oxford Autoimmune Neurology Group, Nuffield Department of Clinical Neurosciences, University of Oxford, Oxford, UK; Department of Clinical Neurosciences, Karolinska Institutet, Stockholm, Sweden; Oxford Autoimmune Neurology Group, Nuffield Department of Clinical Neurosciences, University of Oxford, Oxford, UK; UOC Neurologia, Fondazione Policlinico Universitario A. Gemelli IRCCS, Rome, Italy; Wolfson Centre for Mathematical Biology, Mathematical Institute, University of Oxford, Oxford, UK; Department of Radiology, John Radcliffe Hospital, Oxford University Hospitals, Oxford, UK; Department of Radiology, John Radcliffe Hospital, Oxford University Hospitals, Oxford, UK; Oxford Autoimmune Neurology Group, Nuffield Department of Clinical Neurosciences, University of Oxford, Oxford, UK; Oxford Autoimmune Neurology Group, Nuffield Department of Clinical Neurosciences, University of Oxford, Oxford, UK; Translational Histopathology Laboratory, Department of Oncology, University of Oxford, Oxford, UK; Translational Histopathology Laboratory, Department of Oncology, University of Oxford, Oxford, UK; Translational Histopathology Laboratory, Department of Oncology, University of Oxford, Oxford, UK; Translational Histopathology Laboratory, Department of Oncology, University of Oxford, Oxford, UK; Department of Gynaecologic Oncology, Oxford University Hospitals, Oxford, UK; Department of Animal Model Development, Brain Research Institute, Niigata University, Niigata, Japan; UOC Neurologia, Fondazione Policlinico Universitario A. Gemelli IRCCS, Rome, Italy; Università Cattolica del Sacro Cuore, Rome, Italy; Kids Neuroscience Centre, Children’s Hospital at Westmead, Faculty of Medicine and Health, University of Sydney, Sydney, Australia; University Department of Psychiatry, University of Oxford, Oxford, UK; University Department of Psychiatry, University of Oxford, Oxford, UK; University Department of Psychiatry, University of Oxford, Oxford, UK; Computational, Cognitive and Clinical Neuroimaging Laboratory, Division of Brain Sciences, Imperial College London, London, UK; Department of Neurology, Seoul National University Hospital, Seoul, South Korea; Department of Pathology, Johns Hopkins Hospital, Baltimore, MD, USA; Oxford Autoimmune Neurology Group, Nuffield Department of Clinical Neurosciences, University of Oxford, Oxford, UK; University Department of Psychiatry, University of Oxford, Oxford, UK; Department of Pathology, John Radcliffe Hospital, Oxford University Hospitals, Oxford, UK; Department of Radiology, John Radcliffe Hospital, Oxford University Hospitals, Oxford, UK; Oxford Autoimmune Neurology Group, Nuffield Department of Clinical Neurosciences, University of Oxford, Oxford, UK; Oxford Autoimmune Neurology Group, Nuffield Department of Clinical Neurosciences, University of Oxford, Oxford, UK; Department of Neurology, John Radcliffe Hospital, Oxford University Hospitals, Oxford, UK; Oxford Autoimmune Neurology Group, Nuffield Department of Clinical Neurosciences, University of Oxford, Oxford, UK; Department of Neurology, John Radcliffe Hospital, Oxford University Hospitals, Oxford, UK; Oxford Autoimmune Neurology Group, Nuffield Department of Clinical Neurosciences, University of Oxford, Oxford, UK; Department of Neurology, John Radcliffe Hospital, Oxford University Hospitals, Oxford, UK

**Keywords:** NMDAR-antibody encephalitis, brain autoimmunity, germinal centre, cervical lymph node, teratoma

## Abstract

Autoantibodies against the extracellular domain of the *N*-methyl-d-aspartate receptor (NMDAR) NR1 subunit cause a severe and common form of encephalitis. To better understand their generation, we aimed to characterize and identify human germinal centres actively participating in NMDAR-specific autoimmunization by sampling patient blood, CSF, ovarian teratoma tissue and, directly from the putative site of human CNS lymphatic drainage, cervical lymph nodes. From serum, both NR1-IgA and NR1-IgM were detected more frequently in NMDAR-antibody encephalitis patients versus controls (both *P* < 0.0001). Within patients, ovarian teratoma status was associated with a higher frequency of NR1-IgA positivity in serum (OR = 3.1; *P* < 0.0001) and CSF (OR = 3.8, *P* = 0.047), particularly early in disease and before ovarian teratoma resection. Consistent with this immunoglobulin class bias, ovarian teratoma samples showed intratumoral production of both NR1-IgG and NR1-IgA and, by single cell RNA sequencing, contained expanded highly-mutated IgA clones with an ovarian teratoma-restricted B cell population. Multiplex histology suggested tertiary lymphoid architectures in ovarian teratomas with dense B cell foci expressing the germinal centre marker BCL6, CD21^+^ follicular dendritic cells, and the NR1 subunit, alongside lymphatic vessels and high endothelial vasculature. Cultured teratoma explants and dissociated intratumoral B cells secreted NR1-IgGs in culture. Hence, ovarian teratomas showed structural and functional evidence of NR1-specific germinal centres. On exploring classical secondary lymphoid organs, B cells cultured from cervical lymph nodes of patients with NMDAR-antibody encephalitis produced NR1-IgG in 3/7 cultures, from patients with the highest serum NR1-IgG levels (*P* < 0.05). By contrast, NR1-IgG secretion was observed neither from cervical lymph nodes in disease controls nor in patients with adequately resected ovarian teratomas. Our multimodal evaluations provide convergent anatomical and functional evidence of NMDAR-autoantibody production from active germinal centres within both intratumoral tertiary lymphoid structures and traditional secondary lymphoid organs, the cervical lymph nodes. Furthermore, we develop a cervical lymph node sampling protocol that can be used to directly explore immune activity in health and disease at this emerging neuroimmune interface.

## Teaser

Al-Diwani *et al.* provide anatomical and functional evidence of NMDAR-autoantibody production from active germinal centres within human cervical lymph nodes and ovarian teratomas, using multimodal analyses of tissues obtained directly from patients with NMDAR-antibody encephalitis.

## Introduction

Pathogenic autoantibodies of the IgG isotype that target the extracellular aspect of the NR1 subunit of the *N*-methyl-d-aspartate receptor (NMDAR) cause a multistage encephalitis, predominantly in young females.^1–4^ This prototypical autoantibody-mediated disease of the CNS, a common established cause of encephalitis,^5,6^ often necessitates prolonged hospitalizations and multiple immunotherapies. Nevertheless, despite improvements with treatments, nearly all patients show incomplete clinical responses, and relapses are well-recognized.^1,2,7,8^ A rational approach to address these unmet medical needs is to better understand the key underlying immunological mechanisms focused on the generation of NR1-autoreactive B cells.^9^

In NMDAR-antibody encephalitis, one fundamental immunological insight comes from studies of ovarian teratomas (OT), a characteristic tumour found in around one-quarter of patients.^1,2,10,11^ OT removal expedites patient recovery^2,7^ and its histology reveals dense aggregates of T, B and plasma cells, typically found in close apposition to neuroglial tissue.^10–16^ Collectively, these findings may represent OT-based ectopic germinal centres (GCs),^11,14,15^ a potential site of the autoimmunization.

GCs are traditional sites of immunoglobulin (Ig) affinity maturation and diversification.^17^ The key GC-based process of somatic hypermutation relies on the enzyme activation-induced cytidine deaminase (AID) and is traditionally thought to mature the B cell receptor (BCR) towards a higher affinity. AID is also a key mediator of class-switch recombination,^18^ a DNA excision process that shifts the immune response from early antigen-specific IgMs towards IgA- and/or IgG-isotypes.^19^ This process is relevant to patients with NMDAR-antibody encephalitis as most NR1-directed IgGs are of the IgG1 subclass,^2,10^ although NR1-IgM and NR1-IgA have also been reported.^14,20^ GC B cells express the transcription factor B cell lymphoma 6 protein (BCL6),^17^ and a maturing GC reaction conventionally requires antigen presentation by follicular dendritic cells with B cell help from GC-resident T follicular helper (Tfh) cells.^21^ As both activated Tfh and follicular dendritic cells can release C-X-C motif ligand 13 (CXCL13), this chemokine is considered a marker of GC activity.^22^

While the distinctive association with OTs may offer specific insights into the immunopathogenesis of patients with NMDAR-antibody encephalitis, most patients do not harbour OTs. In these patients, lymph nodes are a more plausible site of GC activity including class-switch recombination and affinity maturation. We proposed that cervical lymph nodes (CLNs) represent the most plausible location for NR1-specific GCs in this condition, as accumulating data indicate meningeal lymphatics drain brain autoantigens into CLNs.^23–25^

Here, to characterize the contributions of secondary and tertiary lymphoid organs in patients with NMDAR-antibody encephalitis, we sample blood and directly access OTs and CLNs, to assess features consistent with NR1-directed GC activity.

## Materials and methods

### Study design

We aimed to study the role of GC activity in the immunopathogenesis of NMDAR-antibody encephalitis with and without associated OTs. Alongside serum and CSF, fresh and fixed surgically resected ovarian tissue and CLN aspirates were studied using a multimodal approach including serology, multi-parameter flow cytometry, lymphocyte culture, single cell RNA sequencing (scRNA-seq), multiplex fluorescence histology and CXCL13 cytokine enzyme-linked immunosorbent assay.

### Patient cohort

For NR1-IgM and -IgA analyses, 285 serum and 60 CSF samples were studied from 108 patients with definite NMDAR-antibody encephalitis (recruited from the Oxford Autoimmune Neurology Group and international centres), defined by CSF NR1-IgG and clinical criteria,^26^ along with 508 sera and 31 CSF from 335 disease and healthy controls (Table 1). As detailed in Supplementary Table 1, a combination of fresh and fixed OT material, was available from four patients who donated five OT samples (one patient had an initial and a rapidly recurrent OT). CLN aspirations were obtained from patients with NMDAR-antibody encephalitis (seven aspirations from six participants, Supplementary Table 1) and 10 comparator participants with other neurological conditions [migraine and antibodies against aquaporin-4 (AQP4), contactin-associated protein 2-like (CASPR2), leucine-rich glioma inactivated 1 (LGI1), glutamic acid decarboxylase and the glycine receptor, Supplementary Table 2].^27^ All samples were obtained with consent in accordance with the 1964 Declaration of Helsinki and ethical approval research ethics committee 16/YH/0013.

**Table 1:** Clinical summaries and serum/CSF samples tested from patients with definite NMDAR-antibody encephalitis and disease controls

	Number of patients	Number of serum samples (first available sample median days from episode onset, range)	Number of CSF samples (first available sample median days from episode onset, range)	Median age (range), years, at disease onset (or time of sampling, for controls)	Female (%)	OT detected (%)
**Cases**						
Definite NMDAR-antibody encephalitis^a^	108	285 (26, 0–3029)	60 (27, 0–579)	20.5 (1–74)	78 (72%)	29 (27%^b^)
**Controls**						
Healthy volunteers	55	55	N/A	36 (20–71)	64	N/A
Traumatic brain injury	58	58	31	39 (21–72)	23	N/A
Bipolar disorder	222	395	N/A	44 (18–62)	54	N/A
**Total controls**	335	508	31	43 (18–72)	50	N/A

N/A = not available.

^a^ All patients had NR1-IgG autoantibodies detectable in CSF and a compatible clinical syndrome.

^b^ No tumours were documented in the male cases.

### Live cell-based assay

Serum and CSF were tested for NR1-specific IgM-, IgG- and IgA-antibodies, with minor modifications to an established live cell-based assay,^2,14^ designed to detect antibodies exclusively against the extracellular domains of the antigenic target. In brief, live human embryonic kidney 293T (HEK293T) cells surface-expressing the NR1 subunit were incubated with serum, CSF or culture supernatant, then washed and fixed, and subsequently incubated with fluorescent secondary commercial antibodies directed against the Fc γ (IgG-specific), Fc α (IgA-specific) or Fc μ (IgM-specific) chains (Jackson laboratories 709-585-098 and 109-585-011, and Invitrogen A21216, respectively). Positive samples were diluted to end point titres. As validated previously,^14^ to confirm isotype specificities, isotype-binding beads were used: Protein G sepharose beads (GE 17-0618-01) for IgG, plus anti-IgM (Sigma 19935) and anti-IgA conjugated agarose beads (Sigma A2691). A cut-off, derived from the median plus the median absolute deviation of control sera, identified a starting dilution of 1:20 as a positivity threshold for each isotype.

### Ultrasound-guided fine needle aspiration and preparation of cervical lymph node material

The nature and possible consequences of the fine needle aspiration procedure were explained through informed consent. An ultrasound scan of the neck including Doppler analysis safely identified accessible CLNs (Siemens Acuson S2000 system and 18L6 HD probe). Then, under sterile conditions, level Va/Vb, or Ia/Ib nodes were targeted via rotatory corkscrew motions of a 23G needle and transferred into Dulbecco’s PBS (D-PBS): the procedure was repeated two to three times. Tuerk’s solution was used to estimate mononuclear cell count and the remaining material was centrifuged at 200*g* for 5 min. The fine needle aspiration pellet was resuspended in D-PBS supplemented with 1% bovine serum albumin, mononuclear cells were separated using Ficoll density gradient solution, washed and then resuspended in D-PBS-1% bovine serum albumin. Aspirate supernatants were removed and stored at −80°C for CXCL13 measurement.

### Multi-parameter flow cytometry

For flow cytometry, cells from dissociated OTs, peripheral blood mononuclear cells (PBMCs), and cells from CLN aspirations were individually labelled at 4°C with a panel of antibodies (Supplementary Table 3). Single stained compensation beads, unstained cells and fluorescence-minus-one controls were used to generate gating and compensation matrices. All analyses were performed on the Attune NxT cytometer (Thermo Fisher). Data analysis and manual gating were performed in FlowJo v9/10 (FlowJo LLC, Becton Dickinson). For the PBMC and CLN analyses, after gating out debris, leucocytes were identified as cells expressing any of CD3, CD14, CD19, CD20, CD27 or CD38. For some experiments, CD45 was included as a confirmatory pan-leucocyte stain, with indistinguishable results. Subsequently, lymphocyte, monocyte, B- and T-cell populations were identified by gating (Supplementary Figs 1 and 2).

### Tumour preparation and histology

For culture, three fresh OT samples were either dissected into 5-mm explants or dissociated with mechanical disruption and brief trypsinization (0.5% trypsin at room temperature). For histology on three OT samples (Supplementary Table 4), commercial antibodies were validated on tonsil tissue with chromogenic and multiplex fluorescent approaches using the Leica BOND RXm autostainer (Supplementary Table 3 and Supplementary Figs 3 and 4). Then, serial 5-μm formaldehyde-fixed paraffin embedded (FFPE) OT sections, and a post-mortem lymph node from a patient with NMDAR-antibody encephalitis,^28^ were stained with the single chromogenic and three multiplex panels (all commercial antibodies listed in Supplementary Table 3). These were digitally scanned using the Vectra Polaris automated quantitative pathology imaging system (Akoya Biosciences) and images extracted using the HALO software package (Indica Laboratories).

### Single cell RNA sequencing

From the OT and paired PBMCs of two NMDAR-antibody encephalitis patients (Supplementary Table 1), B cells (DAPI^−^CD3^−^CD14^−^CD19^+^CD20^+^) were isolated by fluorescence activated cell sorting (Aria III, BD Sciences). Resuspended cells were loaded onto the Chromium 10× platform and 5′ RNA plus BCR sequencing libraries were generated, as per manufacturer guidelines, and sequenced on an Illumina HiSeq4000. Analyses incorporated read alignment and clustering (Cellranger and Seurat, version 3.1.1),^29^ batch effect correction (multi-canonical correlation analysis) and identification/removal of doublets or clusters expressing both T-cell receptor and BCR sequences.^30^ Clustering was performed using a resolution of 0.6 and cluster-specific marker genes identified using FindMarkers in Seurat. Cells were projected into two dimensions using uniform manifold approximation and projection (UMAP) dimensionality reduction. B cell subset identity was annotated by generating synthetic single cell data (500 simulated cells per sample) from B cell reference bulk RNA-seq data^31^ followed by canonical correlation analysis-mediated transfer of labels in Seurat. For visualization, the top quartile of cells by label transfer confidence (defined as the maximum prediction score—the second highest prediction score) were projected into UMAP space. Nearest neighbours plus BCR analysis was undertaken using R with mutational distance from germline estimated using HighVQuest.^32^ BCR sequences were down-sampled 1000 times to the minimum sample size for both patients, and Fisher’s exact test was used to estimate the odds ratio for expanded versus non-expanded clonotypes. The proportion and 95% confidence intervals (CI) of cells expressing each BCR constant region was estimated by the R package metaphor. Enrichment by sample or constant region usage within Seurat clusters was estimated by permuting sample labels 10 000 times. Tissue enrichment was visualized by scaled colouring of each cell’s 200 nearest neighbours.

### Cell culture

Leucocytes were cultured in conditions as described previously.^14^ Briefly, 1 × 10^5^ cells per well were seeded in flat bottom 96-well plates with cytokine-supplemented culture medium, which aimed to either maintain antibody-secreting cells (ASCs) with interleukin (IL)-6 (10 ng/ml; R&D Systems) or—to activate and differentiate B cells into ASCs—additionally with IL-1β (1 ng/ml; PeproTech), IL-2 (50 ng/ml; PeproTech), IL-21 (50 ng/ml; PeproTech), tumour necrosis factor α (TNFα; 1 ng/ml; PeproTech), soluble CD40 ligand (50 ng/ml; R&D Systems) and resiquimod (R848; 2.5 µg/ml; Enzo life sciences). After 13 days *in vitro*, culture supernatants were harvested and stored at −20°C.

### Total IgG and chemokine ligand 13 (CXCL13) enzyme-linked immunosorbent assay

Total IgG levels were determined by enzyme-linked immunosorbent assay from cell culture supernatants (Bethyl Laboratories) and CXCL13 (Quantikine; R&D Systems) levels from both fine needle aspirates and paired sera.

### Statistical analysis

Analyses were performed in Excel (Microsoft), Prism (GraphPad) and R (R Core Team 2017) including extension packages: arm, bayesglm, cellranger, DepecheR,^33^ ggplot2, Seurat,^34^ UMAP and viridis. Proportions were compared using Fisher’s exact test. Paired samples were compared using the Wilcoxon signed-rank test and unpaired using the Mann–Whitney test. The Benjamini–Hochberg procedure was used to correct for multiple comparisons. All comparisons were two-tailed. For the Bayesian general linear model, default values for prior models were used from the bayesglm package. Significance is indicated by asterisks where: *P < 0.05, **P < 0.005 and ***P < 0.0005.

### Data availability

Single cell RNA-seq data have been uploaded to the European Genome-phenome Archive—EGAD00001006232. The other data that support the findings of this study are available from the corresponding author on reasonable request.

## Results

### NR1-specific isotypes associate with ovarian teratoma status

**Figure 1:**
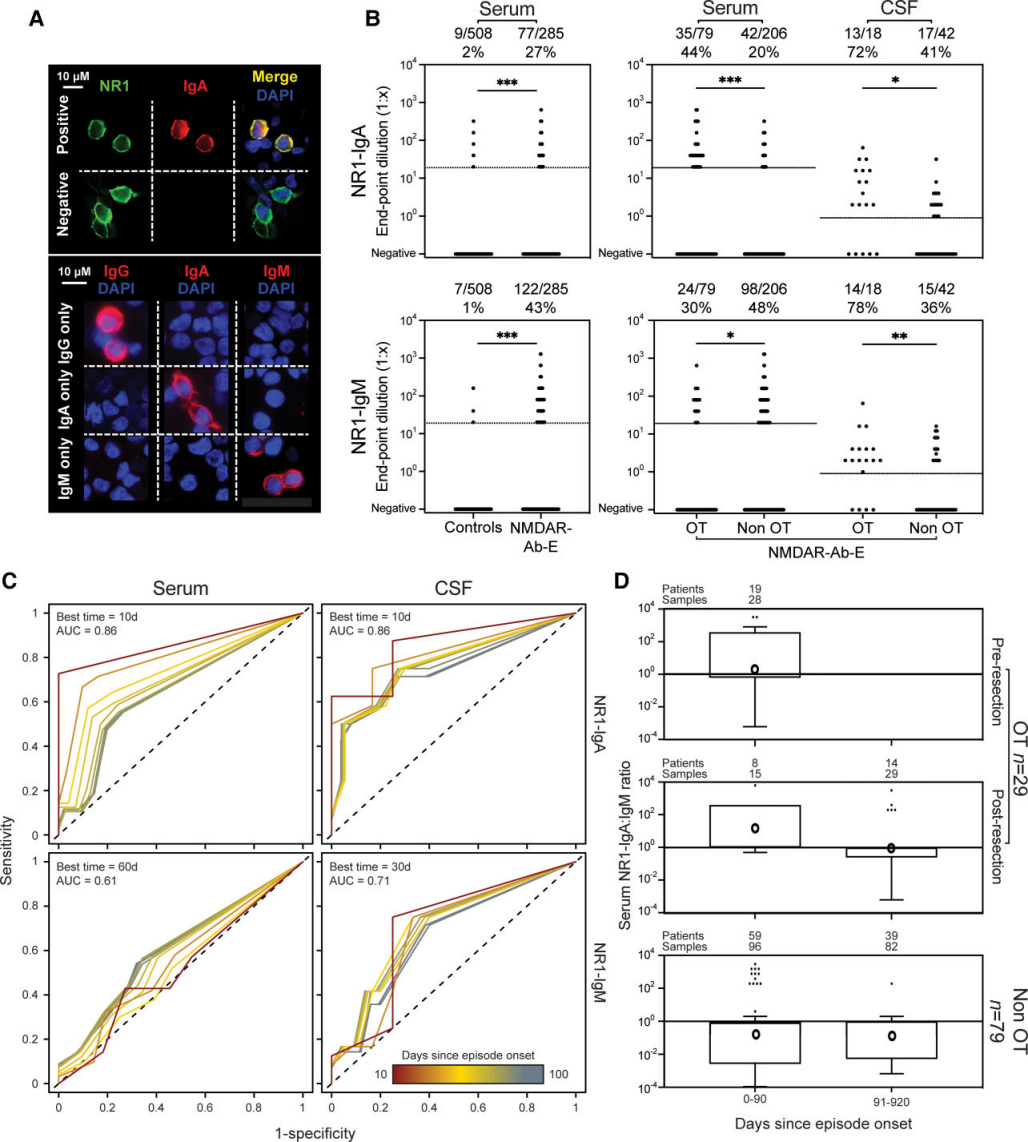
**NR1-directed IgM and IgA antibodies in serum and CSF: association with OT and time from illness onset.** (**A**) Detection of NR1-IgA using a live cell-based assay. *Top*: HEK293T cells were transiently transfected with the NR1 subunit. Expression demonstrated here with an NR1-directed commercial antibody (green). An anti-human IgA Fc specific antibody visualized binding of serum NR1-IgA (Positive); example of a serum sample without NR1-IgA shown (Negative). *Bottom*: After isotype-specific antibody depletion with beads (remaining isotype stated on *left*, white), the detection antibodies used are isotype-specific (detected isotype stated on *top*, red). DAPI to identify HEK293T cell nuclei. (**B**) Frequency and end point dilution of NR1-IgA (*top row*) and NR1-IgM (*bottom row*) in serum and CSF samples from disease/healthy controls (collectively, ‘Controls’) and from patients with NMDAR-antibody encephalitis (NMDAR-Ab-E). Comparisons of serum and CSF from NMDAR-Ab-E patients divided by OT status (OT versus non-OT). Dotted lines indicate positivity cut-off (serum 1:20, CSF undiluted). (**C**) Time-thresholded receiver operating characteristic (ROC) curves for serum or CSF NR1-IgA or NR1-IgM and OT association. The area under the curve (AUC) for serological measures was estimated by ROC analysis. AUC values were calculated for sliding 10-day thresholds between 0 and 370 days since the last episode. The optimum cut-off was assessed by Youden’s J statistic. Ten-day time thresholds from onset to 100 days are shown and indicated by the colour scale (red = earliest, blue = latest). In the *top left* of each box, the time window optimally associated with OT is indicated. Models were unaltered with removal of males. (**D**) Pseudo-log transformed (negative results replaced with 0.1) ratios of serum NR1-IgA:NR1-IgM end point dilutions are plotted over time in patients with and without an associated OT. Samples from OT patients are further subdivided by sampling before or after OT resection. Ratios are plotted on a logarithmic scale in time windows and summarized by box plots using the Tukey method where the horizontal line is the median and individual dots are outliers. Geometric means are indicated by circles. All OT-associated cases underwent OT resection. Upper time limit has been restricted to 920 days post-onset. d = days; DAPI = 4′,6-diamidino-2-phenylindole; NMDAR-Ab-E = NMDAR-antibody encephalitis; OR = odds ratio.

Initially, to identify potential clinical correlates of antigen-specific Ig isotypes, we examined the presence and levels of NR1-IgM and NR1-IgA from longitudinal serum (*n* = 285) and CSF (*n* = 60) samples in 108 patients with NMDAR-antibody encephalitis, by comparison to healthy controls (*n* = 55 patients with 55 serum samples) and disease controls (*n* = 280 patients with 453 serum and 31 CSF samples; Table 1). Live cell-based assay confirmed the co-localization of surface-expressed NR1 with isotype-specific Ig binding, and the expected loss of binding after depletion of specific antibody isotypes (Fig. 1A).^14^ NR1-IgA and NR1-IgM autoantibodies were detected in 77 (27%) and 122 (43%) of the 285 NMDAR-antibody encephalitis sera, respectively, and in only 9/508 (1.7%) and 7/508 (1.3%) samples from disease and healthy control sera (both *P* < 0.0001, two-tailed Fisher’s exact test; Fig. 1B). Within the NMDAR-antibody encephalitis patients, the 79/285 OT-associated serum samples showed a higher frequency of serum NR1-IgA [OR = 3.11 (CI 1.75–5.38), *P* < 0.0001, two-tailed Fisher’s exact test] and less frequent serum NR1-IgM [OR = 0.48 (CI 0.28–0.85), *P* = 0.011, two-tailed Fisher’s exact test; Fig. 1B]. In CSF, both NR1-IgA and NR1-IgM were more frequent in the OT group than the non-OT group [NR1-IgA OR = 3.82 (CI = 1.17–11.46), *P* = 0.047; NR1-IgM OR = 6.30 (CI = 1.77–19.43), *P* = 0.004; Fig. 1B]. Neither NR1-IgA nor -IgM reactivities were detected in CSF from 31 disease controls (Table 1). Using a multivariate model to account for potential sampling confounds between the subgroups including age, time from episode onset, multiple samples from some individual patients and prior immunotherapy associations between OT and NR1-IgA were maintained, but less robustly for NR1-IgM (Supplementary Table 5).

To explore this longitudinally, time-thresholded ROC analyses were generated (Fig. 1C). Earlier times offered the strongest discriminatory value with serum and CSF NR1-IgA positivity showing striking associations with the presence of an OT when sampled within 10 days of symptom onset (AUC = 0.86) and CSF NR1-IgM at 30 days from onset (AUC = 0.71; Fig. 1C). For samples obtained after progressively longer durations from symptom onset (Fig. 1C, heat bar), the AUCs decreased. For example, the serum NR1-IgA association with OT was significantly greater with time thresholds ≤30 days from onset (AUC = 0.80 all samples ≤30 days versus AUC = 0.64 for 40–370 days; *P* = 0.005, Wilcoxon rank sum test). One explanation for these findings was that the presence of an OT promoted class-switch recombination of NR1-antibodies, especially during the earliest disease phase. Concordantly, in the patients with OT, initial serum NR1-IgA:IgM ratios reflected NR1-IgA predominance, a trend that diminished after OT resection (Fig. 1D and Supplementary Fig. 2A). Conversely, in patients without an OT, NR1-IgM dominance persisted for up to 3 years from disease onset despite clinical remission (Fig. 1D). Overall, while NR1-IgM was commonly observed in both OT and non-OT cases of NMDAR-Ab-E, a bias to NR1-IgA was present in patients with OTs.

### Multimodal assessments identify hallmarks of tertiary lymphoid organization in ovarian teratomas

**Figure 2:**
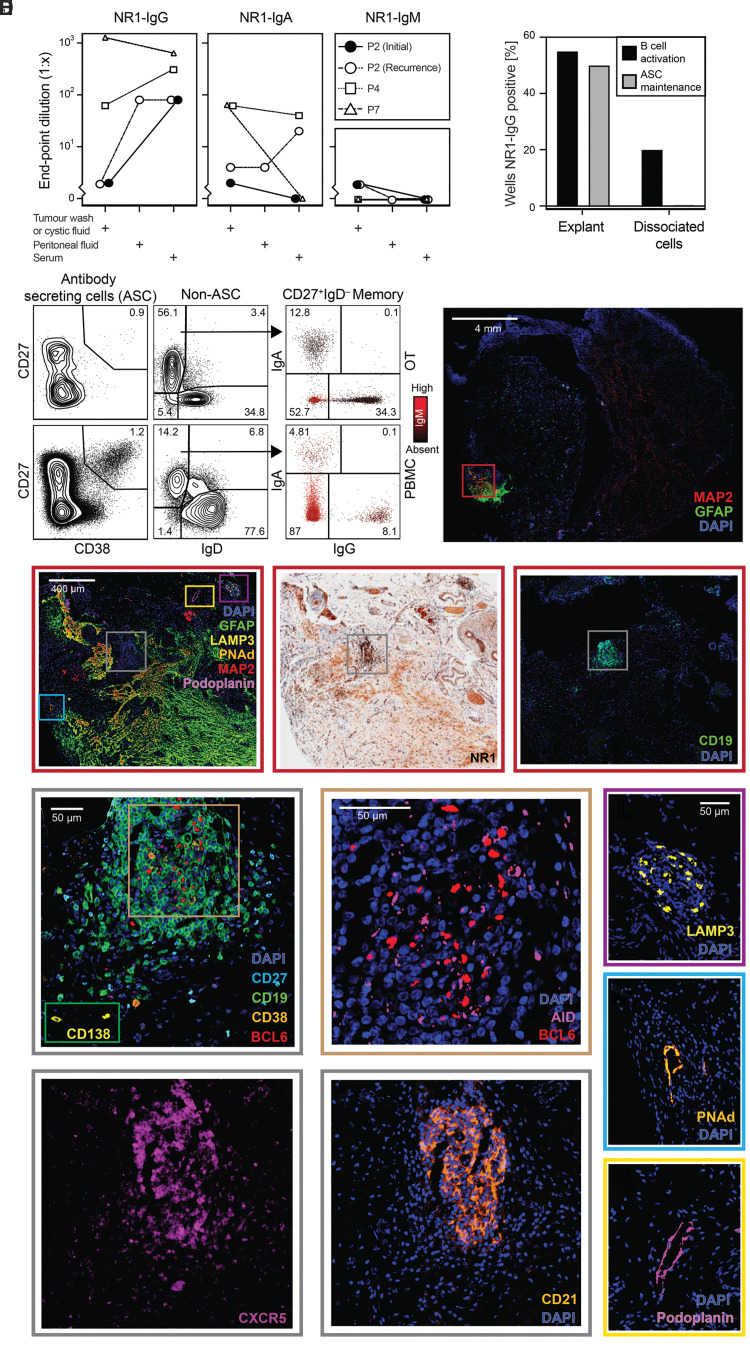
**Structural and functional properties of disease-associated OT:** NR1-autoantibody isotype detection, flow cytometry, cell culture and multiplex histology. (**A**) NR1-antibody isotype levels in matched fluids from four OTs from three patients [indicated by symbols and lines; Patient 2 (P2) had initial and recurrent OTs analysed]. Patient 7 NR1-IgG reproduced here for comparison.^14^ (**B**) B cell populations in the recurrent OT (*top row*) and peripheral blood (PBMCs) of Patient 2. Flow cytometry plots show ASCs (CD19^+^CD27^+^CD38^+^; *left*), naïve (CD27^−^IgD^+^) and memory (CD27^+^IgD^−^) B cells (*middle*) and memory B cell receptor isotypes (IgA versus IgG staining; with IgM staining indicated by colour; *right*). Prior gates shown in Supplementary Fig. 1 (scatter plot lymphocyte gate, doublet exclusion, CD45^+^CD3^−^). (**C**) Frequency of NR1-IgG detection in cell culture supernatants (percentage of wells) from three sets of OT explants and dissociated cells (*right*) using activation (black bars) or maintenance (grey bars) conditions. (**D**) Distributions of lymphocytes, accessory vascular structures, and neuroglial tissue from NMDAR-antibody encephalitis-associated OT tissue (Patient 8). A low power fluorescence image shows a region of co-localized neuronal (MAP2) and glial (GFAP) markers towards the outer aspect of the section (red box). (**E**–**G**) The region of interest is then examined with neuroglial/vascular/dendritic cell markers (**E**), NR1 (**F**) and CD19 (**G**). NR1 staining is most dense in the lymphocytic region (grey box) but can also be seen in the neuroglial region. The CD19^+^ region (grey box and **H**–**K**), dendritic cells (purple box, **L**), and vascular structures (blue and yellow boxes, **M** and **N**, respectively) are then presented. (**H**–**K**) Within the lymphoid aggregate, CD19^+^ cells are densely aggregated with occasional CD38^+^; representative rarer CD138^+^ cells from outside the region are shown in the green *inset* (**H**). Within the centre of the CD19^+^ region (**H**, brown box) there were BCL6^+^ cells, with some cells also positive for AID (**I**). This region also shows CXCR5 expression (**J**) alongside a CD21^+^ meshwork (**K**). (**L**) A cluster of LAMP3^+^ cells consistent with dendritic cells. (**M**) A PNAd^+^ vascular structure consistent with a high endothelial venule. (**N**) A podoplanin^+^ vascular structure consistent with a lymphatic vessel. BCL6 = B cell lymphoma 6 protein; CXCR5 = C-X-C chemokine receptor type 5; DAPI = 4′,6-diamidino-2-phenylindole; GFAP = glial fibrillary acidic protein; LAMP3 = lysosome-associated membrane glycoprotein 3; MBC = memory B cell; P = patient; PNAd = peripheral node addressin.

These findings may indicate differing immunological processes in the OT versus non-OT patients. To address the immunobiology underlying these serological patterns, autoantibodies and lymphocyte populations were examined from the patient OTs (Fig. 2 and Supplementary Table 1). First, one intra-operative peritoneal fluid and four OT-associated fluid samples, were found to contain NR1-IgA in addition to NR1-IgG. Consistent with local intratumoral synthesis, in two of these patients, NR1-IgA was absent in paired serum. By contrast, NR1-IgM was not detected from the sera and cystic fluid and only observed at low levels in two tumour washes (Fig. 2A). Next, dissociated material from three OTs was evaluated by flow cytometry (Supplementary Table 1). Within the intratumoral B cell compartment (CD19^+^CD20^+^; Supplementary Fig. 1), both naïve (CD27^−^IgD^+^) and memory (CD27^+^ IgD^−^) B cells were detected (Supplementary Fig. 2B). Additionally, surface Ig isotypes were studied in one OT demonstrating IgA^+^ and IgG^+^ expressing class switched memory B cells (Fig. 2B). Third, from three fresh OT samples, explants and dissociated cells were cultured under conditions that either activate and differentiate B cells into ASCs or maintain resident ASCs *in vitro* (Fig. 2C). In both culture preparations, NR1-IgGs were secreted under activation conditions, with a higher frequency observed from explant tissue. By contrast, using conditions known to maintain ASCs, NR1-IgG was only generated from explant preparations. In summary, from patients with NMDAR-antibody encephalitis, the detection of intratumoral NR1-specific B cells and ASCs alongside soluble NR1-antibodies are consistent with OTs functioning as active GCs.

To investigate a structural basis for these findings, multiplex immunofluorescence was used to compare the histology of OTs with tissues known to contain GCs, namely tonsil and CLNs.^28^ As expected, organized GCs were observed from surgically resected tonsil tissue (Supplementary Figs 3 and 4A) featuring classical markers of memory B cells (CD19^+^CD27^+^), GC B cells (expressing AID and BCL6) and ASCs (CD19^+^CD27^+^CD38^+^ and CD138^+^). Similar observations were made from CLN tissue (Supplementary Fig. 4B–D). Each of the three studied OTs contained CD19^+^-dense regions that, consistent with previous reports,^11–16^ were often in close proximity to neuronal and glial tissue [microtubule-associated protein 2 (MAP2) and glial fibrillary acidic protein (GFAP), respectively; Fig. 2D and E]. Expression of the NR1-autoantigen co-localized with both the lymphocyte-rich and the MAP2-rich regions (examples in Fig. 2E–G, and Supplementary Figs 5A–F, 6B and 7A). Occasional ASCs were observed and, akin to CLN and tonsil tissue, typically surrounded the CD19^+^ densities (Fig. 2E), which expressed BCL6 (Fig 2H and I and Supplementary Figs 5N and 7B–D). In addition, T cells (CD3^+^ ± CD4^+^) were observed outside the B cell zone (Supplementary Figs 5G and J, 6A and 7B and C). Alongside their expected presence in classical lymphoid vascular structures (Supplementary Figs 3 and 4), lymphatic vessels (demarcated by podoplanin) and high endothelial venules (identified with peripheral node addressin, PNAd), were also seen in OTs (Fig. 2E, M and N and Supplementary Fig. 5H and M). Furthermore, CD19^+^ regions within OTs were associated with a CD21^+^ expressing meshwork, suggestive of professional antigen-presenting follicular dendritic cells, reminiscent of those in archetypal CLN GCs (Fig. 2K and Supplementary Figs 3B, 4D, 5I and 7C).^35^ C-X-C chemokine receptor type 5 (CXCR5), a receptor for CXCL13, was also observed in this region (Fig. 2J and Supplementary Figs 5J and 7C) alongside lysosome-associated membrane glycoprotein 3 (LAMP3) positive dendritic cells (Fig. 2L and Supplementary Figs 3 and 5L). These histological features, analogous to classical lymphoid tissues, support the presence of OT-based GCs.

**Figure 3:**
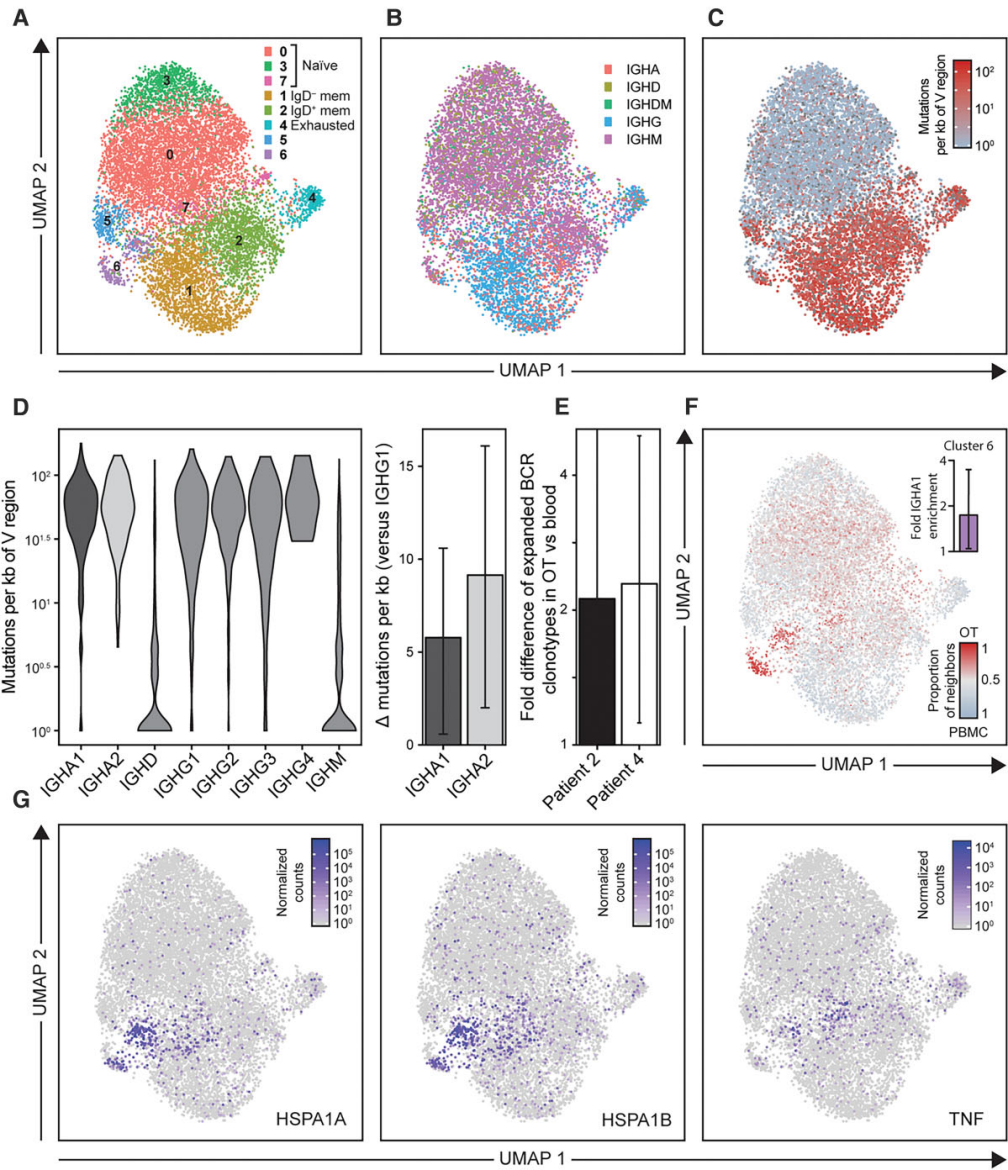
**Single cell RNA sequencing (scRNA-Seq) of peripheral and teratoma-associated B cells**. CD19^+^CD20^+^ cells were sorted from Patient 2 and Patient 4 (initial) teratoma samples and their paired PBMCs. (**A**) UMAP dimensionality reduction plot of scRNA-Seq data from B cells showing cluster identity, using Seurat. Curated cell type identity is labelled. (**B**) B cells coloured by the BCR constant region isotype that is maximally expressed by each cell and (**C**) by the rate of variable region mutations from germline BCR sequences, as estimated by HighVQuest (log_10_ number of mutations per kilobase). This showed a median of 1.65 versus 1.20 log_10_ mutations per kilobase of variable (V) region in a comparison of switched versus unswitched memory B subsets (*P* = 7.49 × 10^–100^). (**D**) Violin plot of variable region mutation rate by BCR constant region and a bar plot of Wilcoxon rank sum differences in the number of mutations per kilobase of V regions between IGHA1/2 and IGHG1 (95% CIs indicated by error bars). (**E**) Bar plot showing the log_2_ fold difference of expanded BCR clonotypes between teratoma and PBMCs (95% CIs based on 1000 downsamplings of the larger set of BCR clonotypes in each case). (**F**) UMAP plot of B cells coloured by *k*-nearest neighbours tissue of origin (*k* = 200) with red representing OT and grey PBMCs. The *inset* shows log_2_ fold enrichment of cells with IGHA1 constant region expression within cluster 6 from 10 000 permutations (*P* = 0.03). (**G**) UMAP plot of B cells coloured by the expression level of top genes enriched within cluster 6. These data have been normalized to the total gene expression, multiplied by 10^4^ and finally 1 has been added to all values, so that 10^0^ equates to an original value of 0. HSP = heat shock protein; IGH = immunoglobulin heavy constant gene; TNF = tumour necrosis factor.

Finally, to study classical GC B cell functions, intratumoral Ig gene mutations and class-switch recombination were explored by comparing BCR sequences between the OTs and matched peripheral blood of two NMDAR-antibody encephalitis patients (Supplementary Table 1). Whole-transcriptome clustering from 10 453 single B cells [with a median of 3289 (IQR 2649–4216) unique molecular identifiers per cell] revealed eight distinct populations (Fig. 3A), including those that correspond to naïve (clusters 0, 3 and 7), exhausted (cluster 4), switched (IgD^−^) memory (cluster 1), unswitched (IgD^+^) memory (cluster 2) and mixed B cells (cluster 5). In both the OT and peripheral B cells, Ig heavy chain constant genes were distributed across B cell subsets as expected (Fig. 3B), and somatic mutations were predominantly observed in memory populations, at greater rates in switched versus unswitched subsets (*P* < 0.0001; Fig. 3C). These data were used to interrogate IgA isotype differences, given the observed association of serum NR1-IgAs with OT status. Interestingly, within OTs the rate of BCR somatic mutations was consistently higher in B cells expressing either IgA1 or IgA2 than in those expressing the known dominant IgG1-subclass of pathogenic NR1-autoantibodies in NMDAR-antibody encephalitis (both *P* < 0.05; Fig. 3D). Also, in a comparison of B cells from OTs versus peripheral blood, although most B cells were found outside of clonally expanded populations there was a significantly higher proportion of expanded BCR clonotypes in OTs [Patient 2: 2.12-fold (CI = 1.41–5.07), *P* < 0.002; Patient 4: 2.29-fold (CI = 1.12–4.99), *P* < 0.002, Fig. 3E]. Finally, to relate this to B cell populations that may be specific to the OT microenvironment, each cluster was analysed by tissue of origin.^36^ Intriguingly, cluster 6 was enriched not only for IgA1-expressing B cells (1.71-fold, 95% CI 1.05–3.5, *P* = 0.03) but also for cells originating from OT samples relative to peripheral blood (Patient 2: 1.68-fold, 95% CI 1.46–1.99, *P* < 0.0002; Patient 4: 1.76-fold, 95% CI 1.32–2.52, *P* < 0.0002; Fig. 3F). Whole-transcriptome analysis of this population revealed increased expression of heat shock proteins (HSPA1A and HSPA1B) and tumour necrosis factor (TNF) (Fig. 3G; *P* < 10^−10^). In summary, scRNA-seq identified intratumoral B cells with evidence of active class switching to IgA that showed higher somatic mutation loads plus clonal expansions, and an OT-specific B cell cluster enriched for expression of IgA and select other molecules.

Taken together, histology, flow cytometry, scRNA-seq and tissue culture provide structural and functional analyses of OTs that converge to identify multiple features of intratumoral ectopic GCs with tertiary lymphoid organization.

### B cells from NMDAR-antibody encephalitis patient cervical lymph nodes carry NR1-reactivities

**Figure 4:**
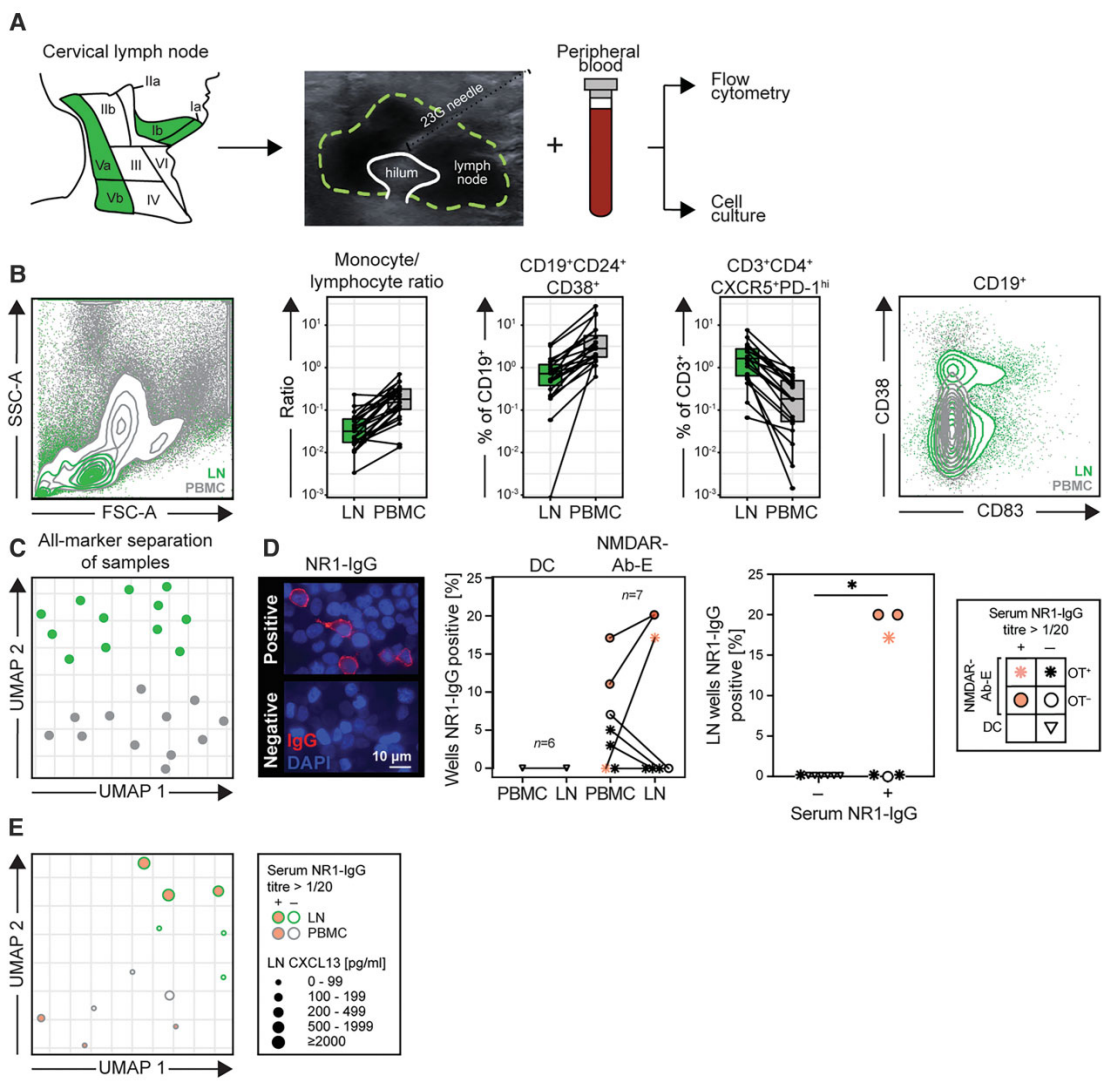
**Evaluation and characteristics of cervical lymph node aspirates.** (**A**) Patient cervical lymph nodes (level Ia, Ib, Va or Vb) were accessed using ultrasound-guided fine needle aspiration. Alongside paired PBMCs, the material was processed for flow cytometry and cell culture experiments. (**B**) Lymphocytes, monocytes and nine B and three T cell populations were manually gated. Statistical comparisons were made for lymph node (LN)-PBMC sample pairs (LN = green, PBMC = grey). *Left*: a representative scatter plot showing superimposed LN and PBMC populations, demonstrating a characteristic absence of granulocyte populations in the LN plot. *Middle*: The three most significantly different parameters (Benjamini–Hochberg corrected Wilcoxon signed-rank tests *P*-values all <0.0001) between LN and PBMC are shown (monocyte:lymphocyte ratio, CD19^+^CD24^+^CD38^+^ transitional B cells and CD3^+^CD4^+^CXCR5^+^PD-I^hi^ T follicular helper cells). *Right*: Flow cytometry plot representative of LN versus PBMC comparisons showing GC B cells (CD19^+^CD83^+^, both with and without CD38) exclusively in CLNs. (**C**) UMAP of all 13 cell subset percentages. Each point represents one sample. (**D**) *Left*: Examples of NR1-IgG positive and negative culture supernatants, using live cell-based assay immunofluorescence. *Middle*: Frequency of cell culture wells with supernatant NR1-IgG reactivities, from paired peripheral blood and lymph node material, in seven samples from patients with NMDAR-antibody encephalitis and six disease controls. LN cultures produced NR1-IgG from samples from Patient 1 (Visits 1 and 2) and Patient 2. *Right*: Frequency of NR1-IgG positivity from LN cultures divided by a routine cut-off (1:20) for serum NR1-IgG end point dilution positivity. Key: Samples with serum NR1-IgG reactivities are coloured pink and patients with OT are indicated by an asterisk; disease control patients are indicated by inverted triangles. (**E**) UMAP of all 13 cell subset percentages from cultured PBMC (grey outline) and LN (green outline) samples. Points are scaled by concentration of CXCL13 detected in paired serum (PBMC samples) or LN aspirate wash (LN samples). CXCL13 = C-X-C motif ligand 13; CXCR5 = C-X-C chemokine receptor type 5; DC = disease control; FSC-A = forward scatter area; G = gauge, NMDAR-Ab-E = NMDAR-antibody encephalitis; PD-1 = programmed death 1; SSC-A = side scatter area.

Most patients with NMDAR-antibody encephalitis do not have OTs. In addition, in patients with active ectopic tertiary lymphoid organs, other sites may contribute towards an autoimmunization. Thus, to investigate the role of classical secondary lymphoid organ resident GCs in NMDAR-antibody encephalitis, we directly sampled patient lymph nodes using ultrasound-guided fine needle aspiration. We focused on the cervical region as it has become increasingly well demonstrated that meningeal lymphatics preferentially drain to CLNs,^24,25^ and therefore represent the most plausible site of autoantigen-specific GC activity in patients with NMDAR-antibody encephalitis (Fig. 4).

From six NMDAR-antibody encephalitis patients and 10 disease controls (Supplementary Tables 1 and 2), paired CLN aspirations and PBMC samples underwent phenotyping by flow cytometry and functional analyses with cell culture (Fig. 4A and Supplementary Fig. 1). A comparison of CLN cells versus PBMCs showed marked differences including raised monocyte:lymphocyte ratios and transitional B cell frequencies (CD19^+^CD24^+^CD38^+^) in PBMCs, versus a prominence of Tfh cells (CD3^+^CD4^+^CXCR5^+^PD-1^hi^) in CLNs (all *P* < 0.0001; Wilcoxon signed-rank tests with Benjamini–Hochberg correction for multiple comparisons; Fig. 4B). Furthermore, CD19^+^ B cells were observed in PBMCs and CLNs, with CD83^+^ GC B cells more prominent in CLNs (Fig. 4B). An unsupervised UMAP incorporating 13 cellular populations showed a striking separation of CLN samples from PBMCs (Fig. 4C and Supplementary Fig. 8), confirming their distinct cellular compositions consistent with recent observations from fine needle aspirations of axillary and inguinal LNs in human volunteers.^37^

This reassuring purity allowed us to ask whether CLNs contain NR1-specific B cells. To investigate this, the activation conditions that successfully generated NR1-IgG from OT B cells were employed.^14,38^ None of the disease control PBMC or CLN samples generated detectable NR1-IgG in culture supernatants (Fig. 4D). By contrast, from NMDAR-antibody encephalitis patients sampled across varied immunotherapy regimes and times since disease onset (Supplementary Table 1), NR1-specific IgG was detected in 5/7 PBMC cultures, in both OT and non-OT patients. However, from CLN cultures, *in vitro* NR1-IgG production was not observed from the three patients with fully resected OTs, but was dectected from 3/7 samples, two from a patient without OT and one patient with a rapidly recurrent OT (Supplementary Fig. 9). Moreover, NR1-IgG was detected in CLN cultures exclusively from participants with higher levels of serum NR1-IgG (*P* < 0.05; Fig. 4D) and trended towards those with the highest CLN levels of an established marker of GC activity, CXCL13 (Fig. 4E and Supplementary Figs 10 and 11).^22^ No differences in total IgG secretion were observed from cultures that did versus did not secrete NR1-IgG (Supplementary Fig. 9).

Taken together, these experiments identify NR1-specific B cells in CLNs exclusively from NMDAR-antibody encephalitis patients, throughout their disease course, particularly from samples that show additional features of ongoing GC activity, but not from patients with adequately resected OTs.

## Discussion

This study has provided evidence across multiple techniques to support the presence of NR1-directed secondary and tertiary lymphoid organ activity in patients with NMDAR-antibody encephalitis. Our findings strengthen both functional and structural evidence that support the role of the OT as an autoimmunizing nidus in this subgroup of patients. Furthermore, we generate the first direct evidence of autoantigen-specific human B cells in the secondary lymphoid tissues most likely to drain the CNS: CLNs. Our study shows that direct analyses of human CLNs can advance our understanding of the CNS-peripheral crosstalk and provide *in vivo* measurements of autoimmunization within organs of great theoretical importance in this process.

We propose that the OT associations of serum and CSF NR1-IgAs reflect active class-switch recombination within the OTs, a hypothesis supported by the loss of NR1-IgA:IgM polarization after OT resection. This reinforces the previous observation of CSF NMDAR-IgAs in association with OT patients,^20^ while introducing serum as a source of potentially similar value. Although these IgM and IgAs may not carry pathogenic potential,^39,40^ their use as biomarkers could offer value in patients with NMDAR-antibody encephalitis found to have radiologically-silent microscopic OTs,^41,42^ particularly as frequencies of both these Igs were low (∼1.5%) across a large and clinically diverse cohorts of control sera.^39,43,44^ More directly, our detection of NR1-IgAs in OT-associated fluid but not in paired sera, together with clonally expanded intratumoral IgA^+^ B cells carrying high levels of somatic mutations, indicate an encapsulated, active, local IgA-promoting OT microenvironment. While class-switch recombination can be a GC-independent process,^45^ OTs also contained several features consistent with prototypical GCs, namely NR1-specific B cells and ASCs,^14^ GC B cells,^11,46^ classical secondary lymphoid organ high endothelial venules and lymphatics, and other key cellular populations that mediate antigen presentation and B cell help, such as Tfh and professional dendritic cells.^11^ In addition, these GCs expressed the autoantigen: consistent with other reports, we observed the NR1 subunit in OT-expressed neuroglial tissue,^46^ and also in lymphocyte-rich regions,^14^ suggesting its expression on diverse cell types may propagate an ongoing GC response. Taken together, these data support an active intratumoral NR1-autoimmunization in patients with NMDAR-antibody encephalitis and strengthen the causal link between OTs and the autoantibody-mediated syndrome,^47^ perhaps explaining the observed clinical benefits from prompt OT resection.^2,7^ It is possible that removal of the identified heat shock protein-expressing B cell population enriched within OTs may be key to clinical recovery. Indeed, heat shock protein 70 (encoded by *HSPA1A* and *HSPA1B*) has been associated with a pro-autoimmune and anti-tumour immune microenvironment, suggesting its potential role in autoantigen-priming within OTs.^48,49^ Our data mean this hypothesis can be directly investigated in future studies.

Of relevance to broader principles regarding CNS autoimmunizations, NR1-specific B cells were detected within CLNs from around half of the NMDAR-antibody encephalitis patient aspirations, but not disease controls. Moreover, the CLN samples that generated NR1-IgG in culture tended towards both higher serum NR1-IgG levels and intranodal CXCL13 levels. Hence, these secondary lymphoid organs with a recently rediscovered role in CNS immunity^24^ show evidence of both ongoing GC activity and NR1-specificity. These observations provide a paradigm to safely study the merits of CLN aspirations in understanding varied forms of CNS autoimmunity and may offer a direct method to test the concept that herpes simplex virus encephalitis can lead to autoantibody-mediated encephalitis via this route of autoimmunization.^50^

Our data challenge the hypothesis that the B cell response is GC-independent in this disease. While one study showed NR1-reactive BCRs from patient CSF have few or no mutations,^51^ potentially contradicting a role for GCs, NR1-specific IgG-expressing B cells were detected exclusively in NMDAR-antibody encephalitis patients without OTs. Others have identified more heavily mutated NR1-specific B cells, albeit fewer, from circulating and intrathecal sources.^52,53^ Future studies should aim to retrieve greater numbers of NR1-specific Ig sequences, from varied patient subgroups, to more definitively establish whether some patients do not rely on GC-based somatic mutations for the generation of pathogenic NR1-specificities. Our observation of frequent serum NR1-IgMs may reflect continual *de novo* GC reactions^14^ that, after class-switch recombination, contribute a source of NR1-IgGs. Alternatively, the NR1-IgMs may represent ongoing activation of preformed IgM-memory B cells or extrafollicular reactions without GC formation.^45^ To develop and validate these observations, future studies should aim to longitudinally evaluate matched PBMCs, OTs and CLNs from larger patient cohorts with untreated first presentations through various disease phases and treatment strategies.

No CLN samples from patients with complete OT resections produced NR1-IgG *in vitro*. Hence, when present, the OT may be the dominant tissue in NR1-specific B cell generation. The only patient with a history of an OT whose CLN generated NR1-IgG in culture re-presented with a symptomatic relapse and proven OT recurrence soon after CLN sampling, only 4 months after her initial ovarian cystectomy. This observation is compatible with OT lymphocytes seeding to distant sites as part of the persistent autoimmunization. It is possible that limiting this spread of NR1-specific cells may underlie the observed time-sensitivity of immunotherapy administration in patients with NMDAR-antibody encephalitis.^7^ Collectively, these findings suggest that our experimental workflow may offer a valuable method to evaluate personalized treatment responses across naturally occurring human CNS autoimmunizations including other autoantibody-mediated diseases, multiple sclerosis and after traumatic brain injury. While our sampling of CLNs was based on the plausible biology of these regions to the CNS, future studies should consider sampling other lymph nodes to determine the specificity of our approach.

In conclusion, these data provide evidence for tissue-compartmentalized GC-driven production of NR1-IgGs in a prototypical autoantibody-mediated human CNS disease. Our findings offer insights that affect understanding pathogenesis and may influence clinical care and trial designs in patients with NMDAR-antibody encephalitis. More broadly, we provide a paradigm to study systemic-CNS immune interactions in humans, in both health and across varied CNS diseases.

## Supplementary Material

awac088_Supplementary_Data

## Abbreviations

ASC: antibody-secreting cell
BCR: B cell receptor
CLN: cervical lymph node
GC: germinal centre
Ig: immunoglobulin
NMDAR: *N*-methyl-d-aspartate receptor
NR1: NMDAR subunit 1
OT: ovarian teratoma
PBMC: peripheral blood mononuclear cell
scRNA-seq: single cell RNA sequencing
UMAP: uniform manifold approximation and projection
