# Supplementary material for: Cervical lymph nodes and ovarian teratomas as germinal centres in NMDA receptor-antibody encephalitis
Source: Brain. 2022 Mar 24;145(8):2742–54. doi: 10.1093/brain/awac088 (PMC9486890; doi:10.1093/brain/awac088)
Supplement: awac088_Supplementary_Data [file awac088_Supplementary_Data.pdf]

Patient	Sex	Age at onset / years	Total IT	Time to 2nd line IT or OT resection / days (treatment)	OT detected	OT CBA	OT FC	OT culture	OT multiplex histology	OT RNA seq	Age at FNA / years	Time from onset to FNA / days (episode; illness)	CLN level	IT at FNA	CLN culture	CLN FC	CLN CXCL13
P1 (FNA 1)	F	30	Pred IVIG PLEX MMF CPM	1729 (CPM)	N	N/A	N/A	N/A	N/A	N/A	35	131; 1743	Ib	Pred CPM	Y	Y	Y
P1 (FNA 2)	F	30	Pred IVIG PLEX MMF CPM	1729 (CPM)	N	N/A	N/A	N/A	N/A	N/A	36	777; 2389	Ia	None	Y	Y	Y
P2* (initial OT)	F	19	Pred PLEX	36 (oophorectomy)	Y	Y (TW)	Y	Y	Y (Sup. Fig. 6)	Y	19	102; 102	Va	Pred	Y	Y	Y
P2* (recurrent OT)	F	19	Pred PLEX	236 (oophorectomy)	Y	Y (TW)	Y	Y	N	N	N/A	N/A	N/A	Pred	N	N	N
P3	F	19	Pred IVIG Aza MMF	None	N	N/A	N/A	N/A	N/A	N/A	37	5198; 6658	Ib	Pred	Y	Y	N
P4	F	32	Pred IVIG PLEX	15 (oophorectomy)	Y	Y (CF)	Y	Y	Y (Sup. Fig. 7)	Y	33	249; 249	Ia	None	Y	N	Y
P5	F	19	Pred PLEX	90 (oophorectomy)	Y	N	N	N	N	N	21	1037; 1037	Ia	None	Y	Y	Y
P6	F	28	Pred PLEX	88 (oophorectomy)	Y	N	N	N	N	N	32	1180; 1180	Ia	None	Y	Y	Y
P7	F	23	Pred PLEX	45 (oophorectomy)	Y	Y (CF)	N	N	N	N	N/A	N/A	N/A	N/A	N	Y	Y

P8	F	17	Pred PLEX	46 (oophorectomy)	Y	N	N	N	Y (Fig. 3, Sup. Fig. 5)	N	N/A	N/A	N/A	N/A	N	N	N
P9	F	18	Pred IVIG PLEX RTX	23 (RTX)	N	N/A	N/A	N/A	N/A	N/A	N/A	N/A	N/A	N/A	N	N	N

Supplementary Table 1. Characteristics of NMDAR-antibody encephalitis patients who underwent ovarian teratoma and/or lymph node sampling.

Abbreviations used: Aza = azathioprine, CBA = cell-based assay, CF = cystic fluid, CPM = cyclophosphamide, d = days, F = female, FC = flow cytometry, FNA = fine needle aspiration, IT = immunotherapy, IVIG = intravenous immunoglobulin, MMF = mycophenolate mofetil, N = no, N/A = not applicable, OT = ovarian teratoma, PLEX = plasma exchange, Pred = prednisolone, RTX = rituximab, TW = tumour wash, Y = yes**;** *OT (initial) was resected via cystectomy prior to FNA but subsequent OT developed (recurrent) and then oophorectomy was performed; others were resected by oophorectomy without recurrence.

Disease	Sex	Age at FNA /years	Total IT	CLN level	Current IT	Culture	Flow cytometry	CXCL13
Migraine	F	31	Nil	Va	Nil	Y	Y	Y
Migraine	M	31	Nil	Va	Nil	Y	N	N
Migraine	M	74	Nil	Ia	Nil	N	N	Y
CASPR2	M	72	Pred PLEX	Ia	Pred	Y	N	N
LGI1	M	62	Pred PLEX	Ia	Nil	Y	Y	N
LGI1	F	72	Nil	Va	Nil	Y	N	N
GlyR	F	61	Pred PLEX	Ib	Pred	Y	Y	Y
GAD	F	68	Pred PLEX IVIG CPM	Ib	Nil	N	N	Y
AQP4	F	50	Pred MMF	Vb	MMF	N	Y	N
AQP4	F	53	Pred MMF	Ia	Pred Aza	N	Y	N

Supplementary Table 2. Characteristics of disease controls participants who underwent cervical lymph node sampling.

Abbreviations used: AQP4 = aquaporin-4 antibody associated neuromyelitis optica, Aza = azathioprine, CASPR2 = Contactin-associated protein-like 2-antibody encephalitis, CLN = cervical lymph node, CPM = cyclophosphamide, F = female, GAD = gluatamic acid decarboxylase antibody associated encephalitis, GlyR = glycine receptor antibody-associated encephalitis, IT = immunotherapy, LGI1 = Leucine-rich glioma-inactivated 1-antibody encephalitis, M = male, MMF = mycophenolate mofetil, N = no, PLEX = plasma exchange, Pred = prednisolone, Y = yes

Target	Clone	Dye	Manufacturer
Flow cytometry - ovarian teratoma phenotyping			
CD3<br>CD14<br>Dead cells	UCHT1<br>HCD14	PB<br>PB<br>DAPI	Biolegend<br>Biolegend
CD27	O323	BV605	Biolegend
CD20	2H7	BV711	Biolegend
IgD	IA6-2	FITC	Biolegend
IgA	IS11-8E10	PE	Miltenyi
IgG	G18-145	PE-CF594	BD
IgM	MHM-88	AF-647	Biolegend
CD45	2D1	AF-700	Biolegend
CD19	SJ25C1	APC-Cy7	BD
Flow cytometry - ovarian teratoma cell sorting			
CD3	UCHT1	PB	Biolegend
CD14	HCD14	PB	Biolegend
CD19	SJ25C1	BV510	BD
CD20	2H7	APC	BD
Flow cytometry – paired lymph node peripheral blood phenotyping			
B cell panel			
Live-Dead	-	DAPI	
CD3	UCHT1	PB	Biolegend
CD14	HCD14	PB	Biolegend
CD24	ML5	BV510	Biolegend
CD27	O323	BV605	Biolegend
IgG	G18-145	BV711	BD
CD20	2H7	FITC	BD
IgD	IA6-2	PE-CF594	BD
CD19	SJ25C1	PE-Cy7	BD
IgM	G20-127	APC	BD
CD45	2D1	AF700	Biolegend
CD38	HB7	APC-Cy7	BD
B cell accessory panel			
Live-Dead	-	DAPI	
CD3	UCHT1	PB	Biolegend
CD14	HCD14	PB	Biolegend
IgD	IA6-2	BV510	Biolegend
CD27	O323	BV605	Biolegend
CD83	HB15e	PE-CF594	Biolegend
CD19	SJ25C1	PE-Cy7	BD
CXCR4	12G5	APC	Biolegend
CD45	2D1	AF700	Biolegend
CD38	HB7	APC-Cy7	BD
T cell panel			
Live-Dead	-	DAPI	
CD3	UCHT1	BV510	BD
CXCR5	J252D4	BV711	Biolegend
PD-1	J105	APC	eBioscience

CD45	2D1	AF700	Biolegend
CD4	RPA-T4	APC-Cy7	Biolegend
Multiplex histology – ovarian teratoma			
B cell panel			
CD19	BT51E	Opal 520	Leica
CD138	MI15	Opal 540	Thermo Fisher
CD38	Polyclonal	Opal 570	Abcam
BCL6	LN22	Opal 620	Leica
AID	ZA001	Opal 650	Thermo Fisher
CD27	EPR8569	Opal 690	Abcam
T cell / Follicular dendritic cell panel			
CD4	4B12	Opal 520	Leica
PD-1	EP197	Opal 540	Cell marque
CXCR5	2G9	Opal 570	Leica
CD3	LN10	Opal 620	Leica
CD21	HPA042432	Opal 650	Atlas
Vascular / Neuro-glial panel			
GFAP	GA5	Opal 520	Leica
LAMP3	HPA051467	Opal 540	Atlas
PNAd	sc-19602	Opal 570	Santa Cruz
MAP2	AMAb91375	Opal 620	Sigma
Podoplanin	GTX31231	Opal 650	GeneTex
CD68	M0876	Opal 690	Dako
NR1 chromogenic single stain			
NR1	1H13L3	-	Thermo Fisher

Supplementary Table 3. Commercial antibodies for flow cytometry and histology experiments.

Patient	Location	Pathologic diagnosis	Macroscopic features	Microscopic features
2 (initial)	Right ovarian cyst	Mature cystic teratoma	Caseous necrotic material and hair.	Cyst lined by mature skin including appendages.
4	Right ovarian cyst	Mature cystic teratoma	Hairs and a tooth like structure.	Cyst lined by keratinising epithelium with skin appendages. Also lined by columnar epithelium, cartilage, glial tissue, and bone. Mature neural tissue seen.
8	Left ovarian cyst	Mature cystic teratoma	Sebaceous material and hair.	Mixed variety of tissue types including mature adipocytes and keratinising squamous epithelium with adnexal structures, including sebaceous glands and hair follicles. Also mucinous glandular type epithelium.

Supplementary Table 4. Histopathological summary of Ovarian teratomas included in multiplex histology.

	NR1-IgA		NR1-IgM		Sex	Age	Time from onset	Prior IT	NR1-IgG
	Univariate OR (95% CI), P	Multivariate ß P	Univariate OR (95% CI), P	Multivariate ß P	Multivariate ß P				
**Serum**									
**All samples**<br>n=108 patients<br>n=285 samples	3.11<br>(1.75-5.38), **P<0.0001**	0.35<br>**P=0.00004**	0.48<br>(0.28-0.85), **P=0.01**	-0.23<br>**P=0.0008**	3.42<br>**P=0.035**	0.01<br>P=0.42	-0.001<br>P=0.58	-0.43<br>P=0.36	0.15<br>P=0.11
**All samples excluding males**<br>n=78 patients<br>n=244 samples	4.91<br>(2.58-9.34), **P<0.0001**	0.35<br>**P=0.00004**	0.43<br>(0.24-0.76), **P=0.004**	-0.24<br>**P=0.0008**	N/A	0.02<br>P=0.37	-0.001<br>P=0.58	-0.47<br>P=0.32	0.15<br>P=0.11
**Earliest sample**<br>n=108 patients and samples	3.87<br>(1.58-9.91), **P=0.004**	0.30<br>**P=0.02**	1.14<br>(0.59-3.43), P=0.51	-0.04<br>P=0.71	3.09<br>P=0.053	0.02<br>P=0.58	0.009<br>P=0.096	-1.57<br>P=0.075	0.14<br>P=0.44
**Earliest sample excluding males**<br>n=78 patients and samples	6.56<br>(2.41-18.54), **P=0.0003**	0.29<br>**P=0.02**	1.03<br>(0.43-2.51), P=1	-0.039<br>P=0.73	N/A	0.02<br>P=0.48	0.009<br>P=0.096	-1.62<br>P=0.068	0.14<br>P=0.44
**CSF**									
**All samples**<br>n=60 patients<br>n=60 samples	3.82<br>(1.17-11.46), **P=0.047**	0.54<br>P=0.05	6.30<br>(1.77-19.43), **P=0.004**	0.02<br>P=0.94	2.98<br>P=0.061	-0.05<br>P=0.29	0.004<br>P=0.49	-1.26<br>P=0.17	-0.008<br>P=0.97
**All samples excluding males**<br>n=40 patients<br>n=40 samples	5.57<br>(1.49-22.92), **P=0.025**	0.55<br>P=0.05	4.20<br>(1.09-14.14), P=0.055	0.01<br>P=0.97	N/A	-0.04<br>P=0.31	0.004<br>P=0.47	-1.32<br>P=0.16	-0.004<br>P=0.98

Supplementary Table 5. Univariate and multivariate analyses of serum and CSF NR1-IgA and NR1-IgM association with OT. Univariate odds ratios compared with multivariate statistics in differing sub-groups of serum and CSF samples for the two factors of interest, NR1-IgA and -IgM, and multivariate statistics for possible confounding variables. Multivariate analysis was performed using a Bayesian general linear model on pseudo-log transformed titres. One CSF sample available per patient, all are the earliest available sample.

Abbreviations used: CI = confidence interval, CSF = cerebrospinal fluid, OR = odds ratio

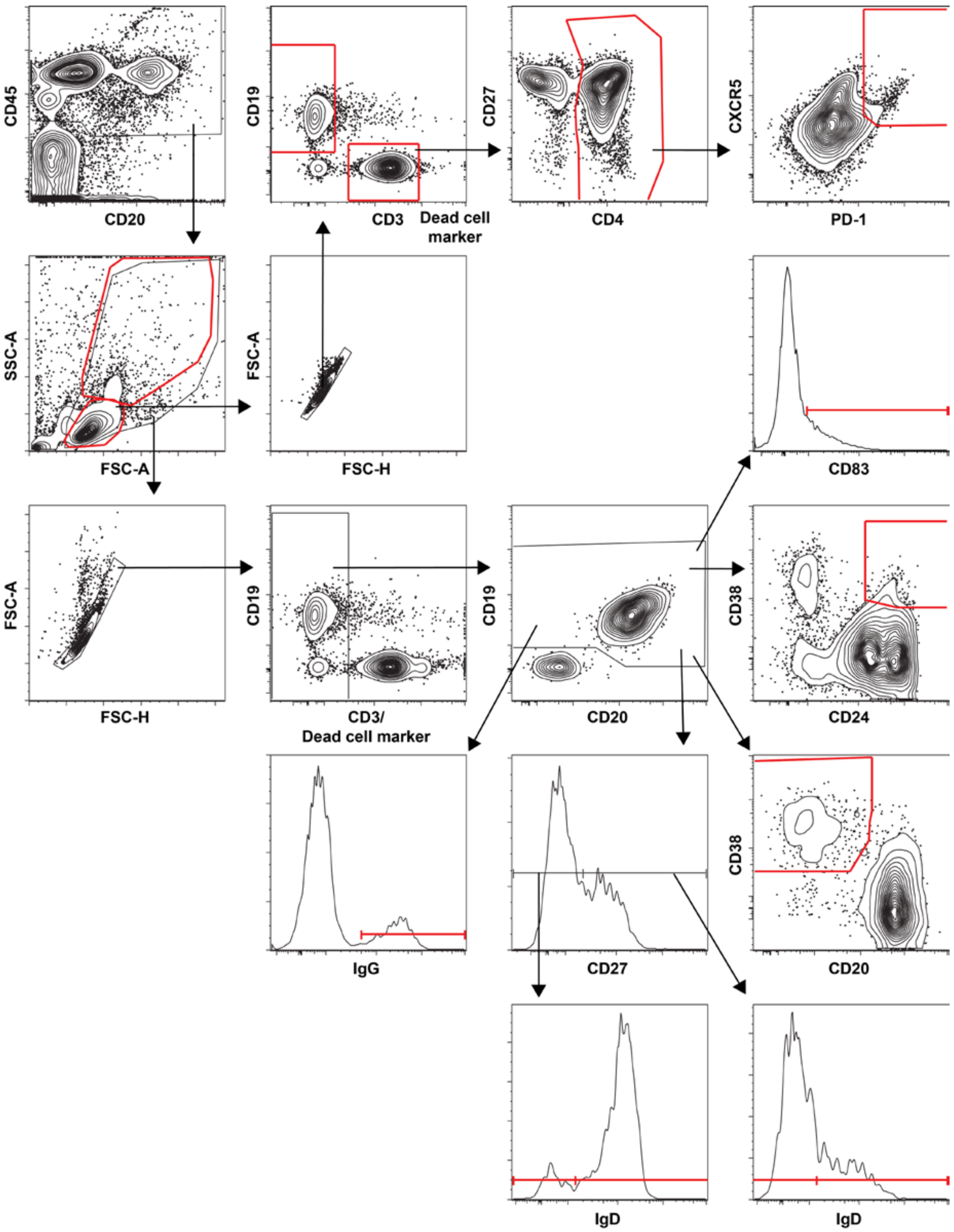

Supplementary Figure 1. Gating strategy for T follicular helper and B cell subsets General gating strategy for the flow cytometry data analysis. Red gates highlight populations that were analysed and used to construct UMAPs.

Abbreviations used: A = area, CXCR5 = C-X-C chemokine receptor type 5, FSC = forward scatter, H = height, Ig = immunoglobulin, PD-1 = programmed death 1, SSC = side scatter, UMAP = uniform manifold approximation and projection (for dimension reduction)

A

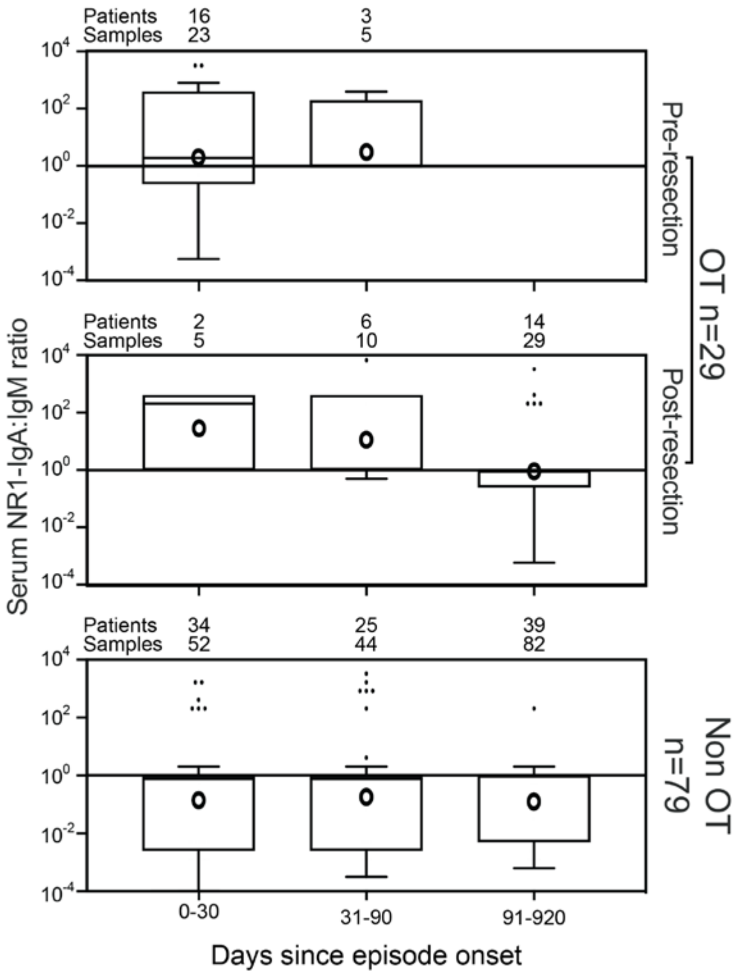

B

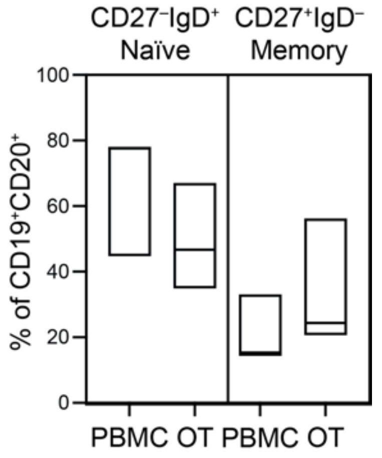

Supplementary Figure 2. Additional data to accompany Figure 1 and 2. (A) As per Fig. 1D, pseudo-log transformed (negative results replaced with 0.1) ratios of serum NR1-IgA:NR1-IgM end-point dilutions are plotted over time in patients with and without an associated OT. Here, ratios are plotted in three time windows summarised as boxplots. The Tukey method is used where the horizontal line is the median and individual dots are outliers. Geometric means are indicated by circles. (B) Flow cytometry of ovarian teratoma cells from patient 2 (initial OT) and patient 4. Frequency of naïve (IgD^+CD27^-) and memory B cells (IgD^-CD27^+) as proportion of total $CD19^+CD20^+$ B cells in ovarian teratoma material versus peripheral blood (median and interquartile range shown).

Abbreviations used: PBMC = peripheral blood mononuclear cell, OT = ovarian teratoma

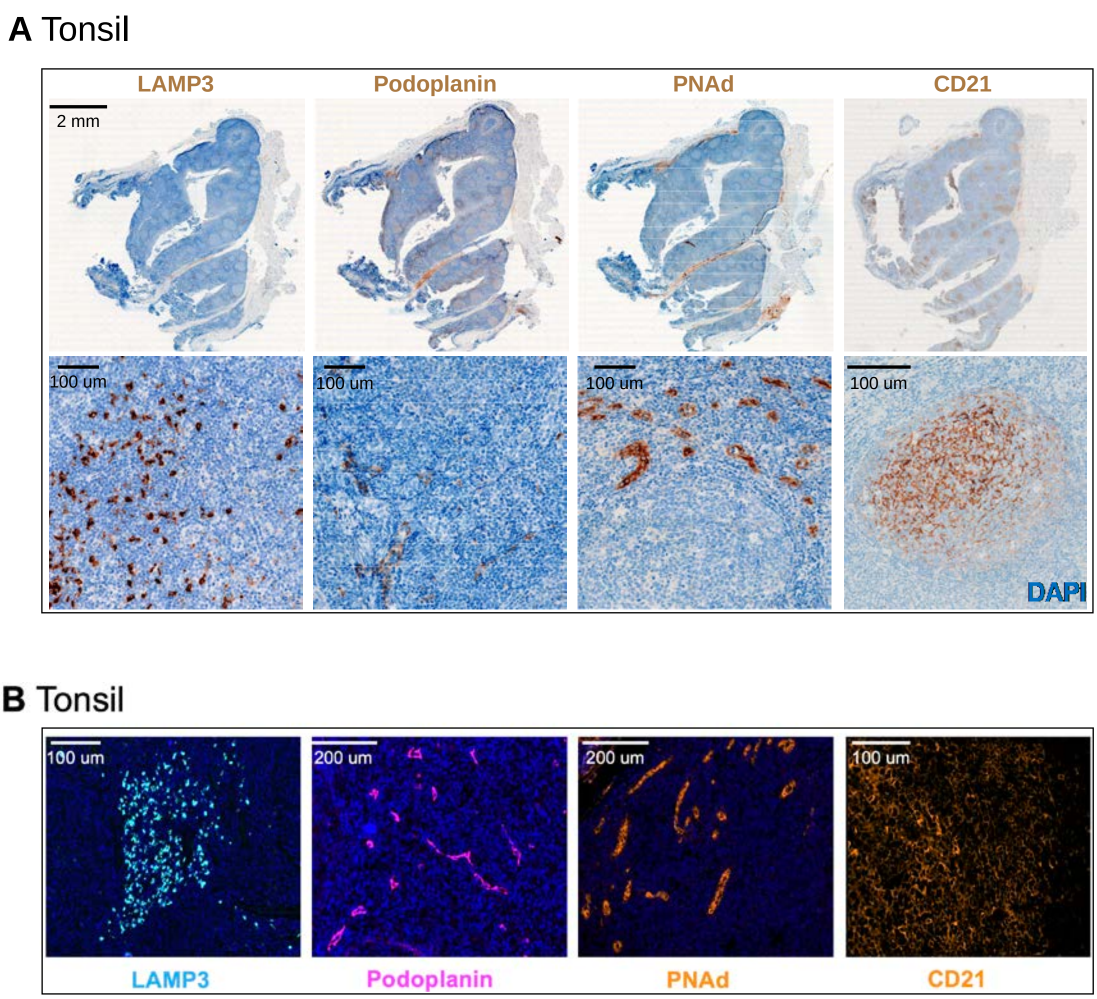

Supplementary Figure 3. Validation of individual antibodies using tonsil tissue (A) Chromogenic staining appearances of LAMP3, Podoplanin, PNAd, and CD21 on control tonsil tissue at low power (top row) and at higher power magnification (bottom row). (B) Multiplex immunofluorescent appearances of corresponding markers from A. Scale bars indicated within.

Abbreviations used: DAPI = 4′,6-diamidino-2-phenylindole, LAMP3 = lysosome-associated membrane glycoprotein 3, PNAd = peripheral node addressin

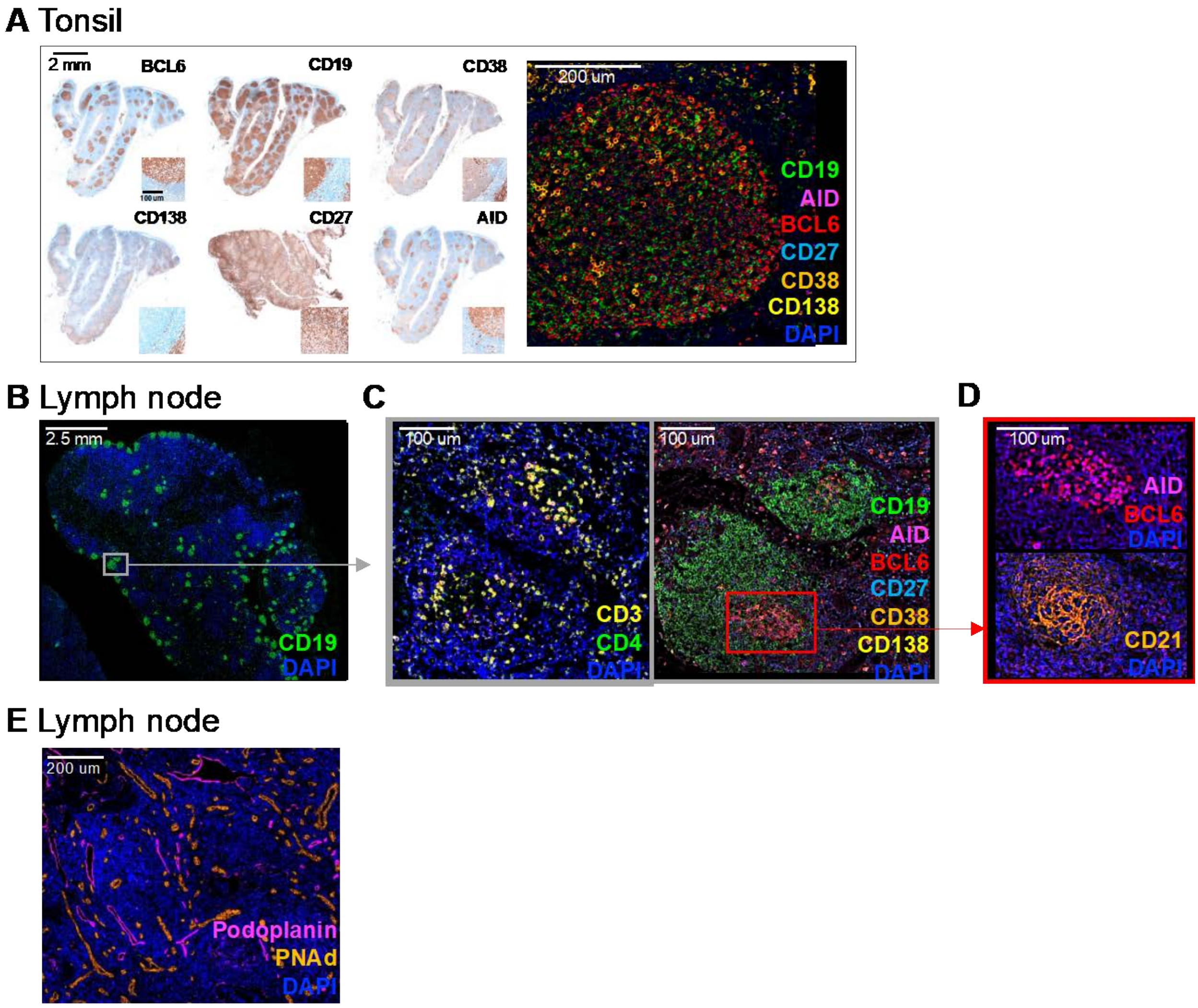

Supplementary Figure 4. Validation of antibodies, individually and in multiplex, on tonsil and lymph node (A) B cell multiplex staining on tonsil. Single markers with chromogenic staining on serial sections (left) and together, in fluorescence multiplex within a single section (right). (B) Low power magnification immunofluorescence image of post-mortem cervical lymph node from a patient with treatment-resistant NMDAR-antibody encephalitis staining for B cells with CD19 (see Nauen et al. 2016). The node itself was not histologically abnormal so used as a comparator of typical germinal centre organisation. A pair of $CD19^+$ regions are then shown in more detail (grey box). (C) Region of interest at higher power showing $CD3^+$ T cells (left) and $CD19^+$ B cells with other markers of differentiation (CD27,38,138) (right). A core of cells expressing AID and/or BCL6 is identified by the red box. (D) High power image of AID and/or BCL6 expression within the germinal center core (top) and associated CD21 expression from a sustentacular meshwork consistent with follicular dendritic cells (bottom). (E) Frequent vascular structures were identified, shown in cross section to express podoplanin (lymphatic vessels) and PNAd (high endothelial venules). Scale bars indicated within.

Abbreviations used: AID = activation-induced cytidine deaminase, BCL6 = B-cell lymphoma 6 protein, DAPI = 4′,6-diamidino-2-phenylindole, PNAd = peripheral node addressin

Ovarian teratoma

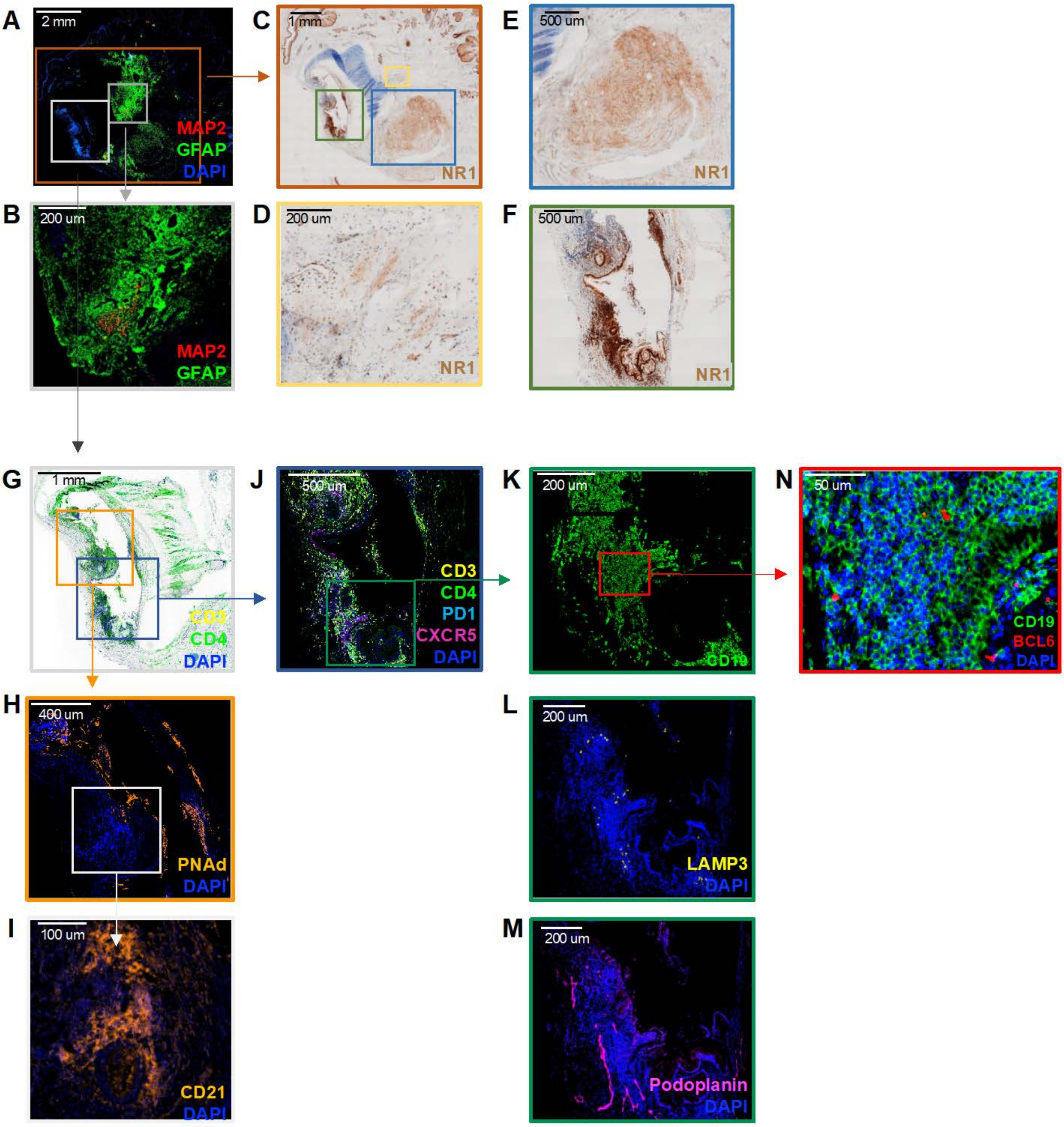

Supplementary Figure 5. Photomicrographs of ovarian teratoma associated germinal center organization from patient 2 initial teratoma (A-F) Multiplex immunofluorescence and chromogenic images showing relationships between neuroglial ($GFAP^+$/$MAP2^+$) and lymphocytic regions. (A) Low power image showing neuro-glial staining. (B) Detail from region of prominent GFAP staining showing a region of MAP2 staining within [grey box from A]. (C) Low power image showing NR1 staining [orange box from A]. Regions of interest are presented in D-F. (D) NR1 staining in the $GFAP^+$ and $GFAP^+MAP2^+$ region shown in B [yellow box from C]. (F) NR1 staining in the region of lymphocytic infiltration presented in G-N. The staining pattern is distinct in morphology and intensity from the neuro-glial region. (G-N) T and B cell staining in region of lymphocytic infiltration. (G) Low power inverted fluorescence image showing CD3 and CD4 staining [light grey box from A]. (H) Detail from g showing PNAd staining consistent with high endothelial venules [orange box from G]. (I) $CD21^+$ cells arranged as a meshwork suggestive of follicular dendritic cells [white box from H]. (J) Detail of T cell staining showing PD1 and CXCR5 staining [blue box from G]. (K) Region of T cell infiltration also containing B cells [green box from J] (L) $LAMP3^+$ staining within the T cell region suggestive of dendritic cells. (M) $Podoplanin^+$ vascular structures within the T cell region suggestive of lymphatic vessels. (N) Detail from B cell regions showing scattered cells expressing BCL6 [red box from K]. Scale bars indicated within.

Abbreviations used: BCL6 = B-cell lymphoma 6 protein, CXCR5 = C-X-C chemokine receptor type 5, DAPI = 4′,6-diamidino-2-phenylindole, GFAP = glial fibrillary acidic protein, LAMP3 = lysosome-associated membrane glycoprotein 3, MAP2 = microtubule-associated protein 2, NR1 = N-methyl-d-aspartate receptor subunit 1, PD1 = programmed death 1, PNAd = peripheral node addressin

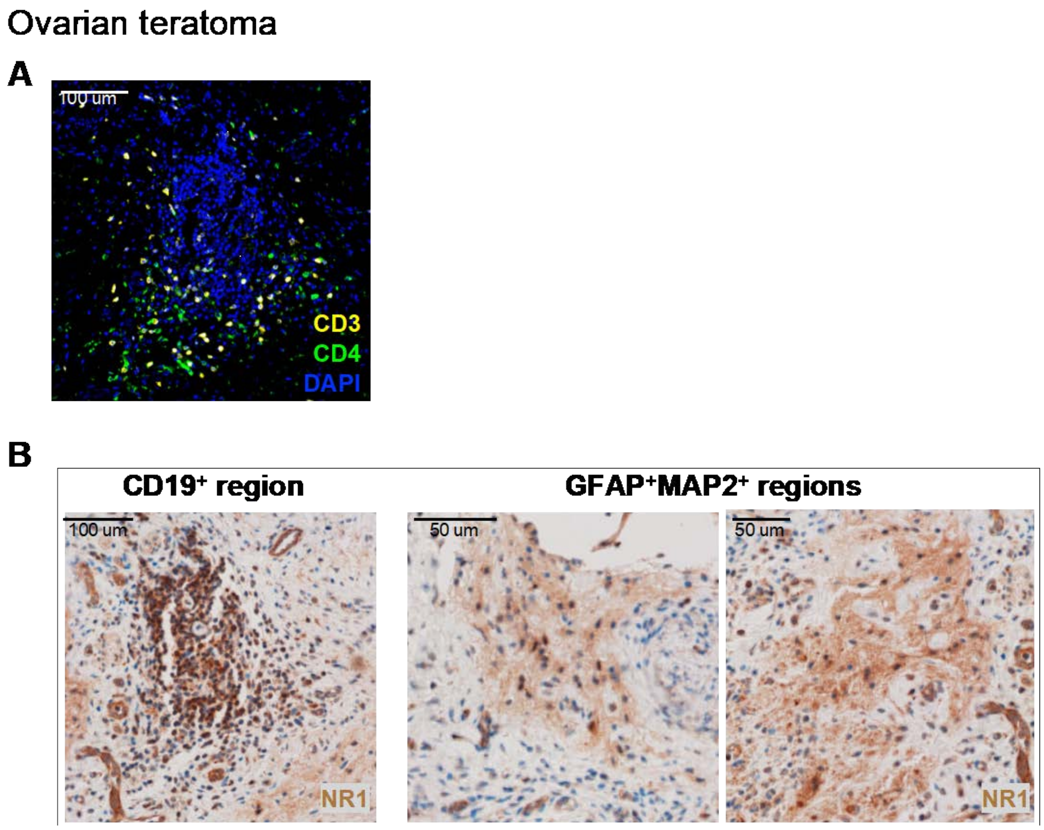

Supplementary Figure 6. Additional photomicrographs of ovarian teratoma associated germinal center organization and relationship with NR1 staining from patient 8 teratoma (A) Multiplex T cell panel images from lymphocytic region shown in Fig. 2. (B) NR1 staining in the CD19^{+} lymphocytic region and the GFAP^{+}/MAP2^{+} neuro-glial region. Scale bars indicated within.

Abbreviations used: DAPI = 4′,6-diamidino-2-phenylindole, GFAP = glial fibrillary acidic protein, MAP2 = microtubule-associated protein 2, NR1 = N-methyl-d-aspartate receptor subunit 1

Ovarian teratoma

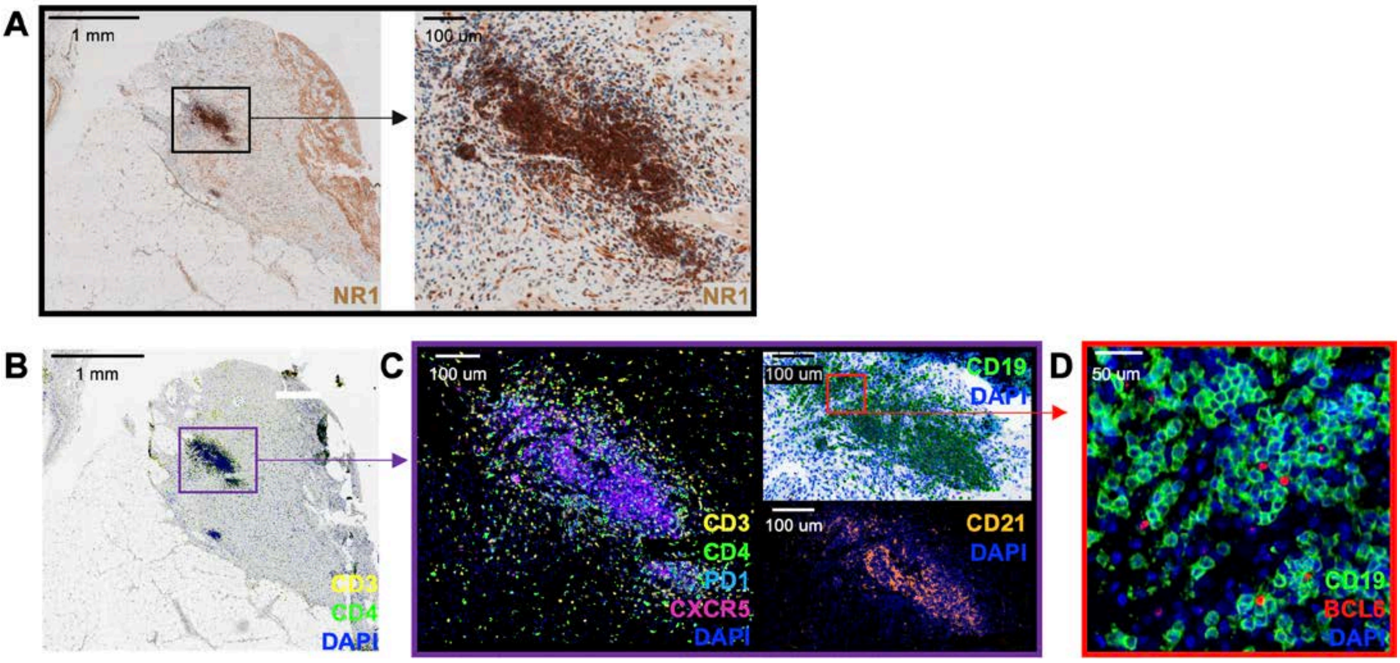

Supplementary Figure 7. Additional photomicrographs of ovarian teratoma associated germinal center organization from patient 4 teratoma (A) Serial section corresponding to a showing NR1 chromogenic staining which co-localizes with lymphocytes but also non-lymphocytic staining nearby. (B) Low power inverted multiplex immunofluorescence image showing a region of lymphocytic infiltration. (C) Detail from lymphocytic region showing T cell distribution on the margins (left; purple box from B) of CD19+ B cell core (top right) which sit within a $CD21^{+}$ mesh consistent with follicular dendritic cells (bottom right). (D) Detail from $CD19^{+}$ region showing intracellular BCL6 staining (red box from C). Scale bars indicated within.

Abbreviations used: BCL6 = B-cell lymphoma 6 protein, CXCR5 = C-X-C chemokine receptor type 5, DAPI = DAPI = 4′,6-diamidino-2-phenylindole, NR1 = N-methyl-d-aspartate receptor subunit 1, PD1 = programmed death 1.

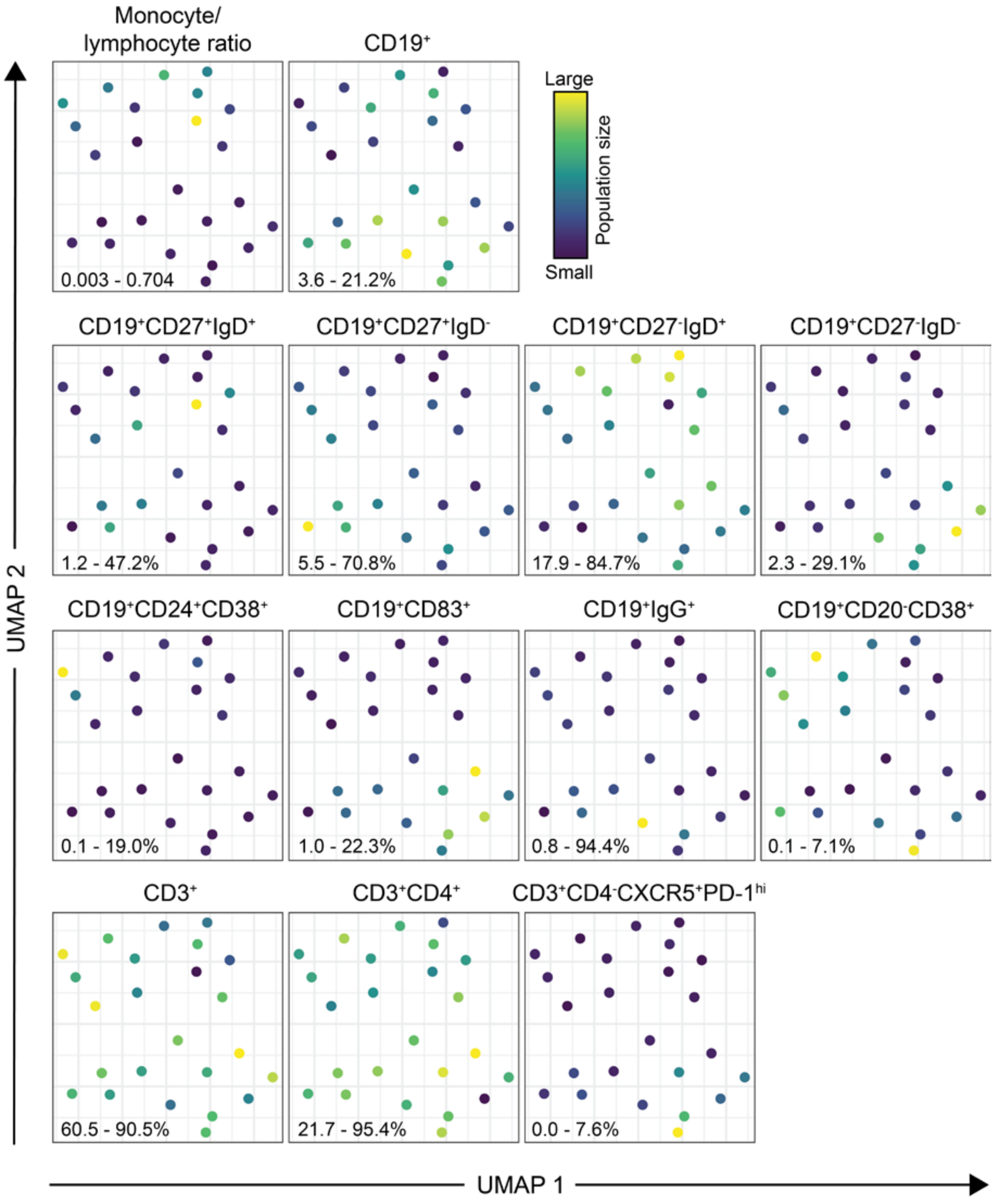

Supplementary Figure 8. The distribution of individual population sizes over UMAP field with relation to Fig. 4C. The twelve populations and the monocyte/lymphocyte ratio used to construct the UMAP are displayed individually over the UMAP field. Each dot represents one sample. Colors denote large (yellow) to small (blue) relative percentages of each leucocyte population. $CD3^+$ and $CD19^+$ percentages are based on confirmed lymphocyte singlet population size.

Abbreviations used: CXCR5 = C-X-C chemokine receptor type 5, Ig = immunoglobulin, PD-1 = programmed death 1, UMAP = uniform manifold approximation and projection for dimension reduction

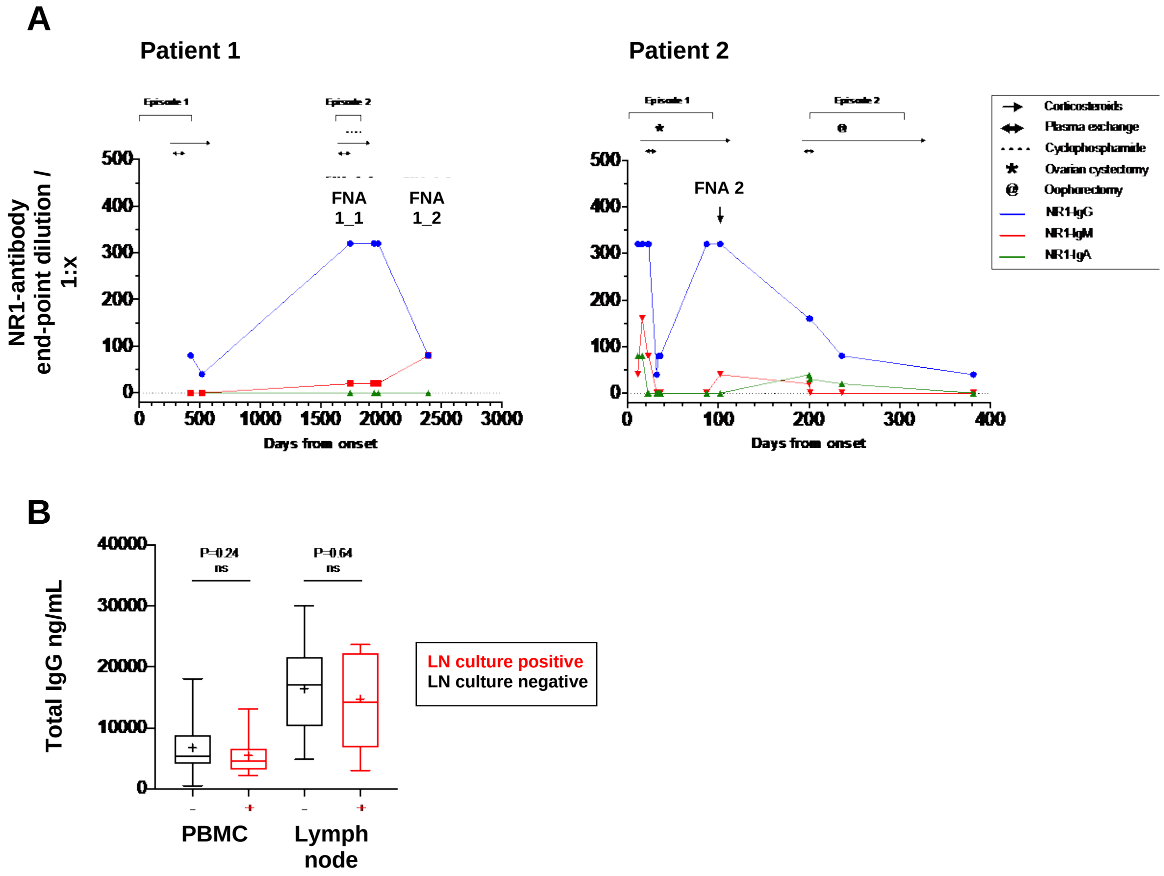

Supplementary Figure 9. Additional data relating to Figure 4. (A) Fine needle aspiration timings in relation to serological time course from two patients where lymph node cells produced NR1-IgG in culture. Episodes of acute symptoms are indicated alongside immunotherapies given (see key). (B) Total IgG detected from unfractionated PBMC and LN cultures showed no significant difference between wells whose supernatant was positive for NR1-IgG versus negative. Horizontal line indicates median, plus sign indicates mean, box is interquartile range, and whiskers are upper and lower values. There was no significant difference between NR1-IgG culture positive or negative sub-groups of PBMC and LN cultures (Mann-Whitney test).

Abbreviations used: FNA = fine needle aspiration, Ig = immunoglobulin, LN = lymph node, NR1 = N-methyl-d-aspartate receptor subunit 1, PBMC = peripheral blood mononuclear cells.

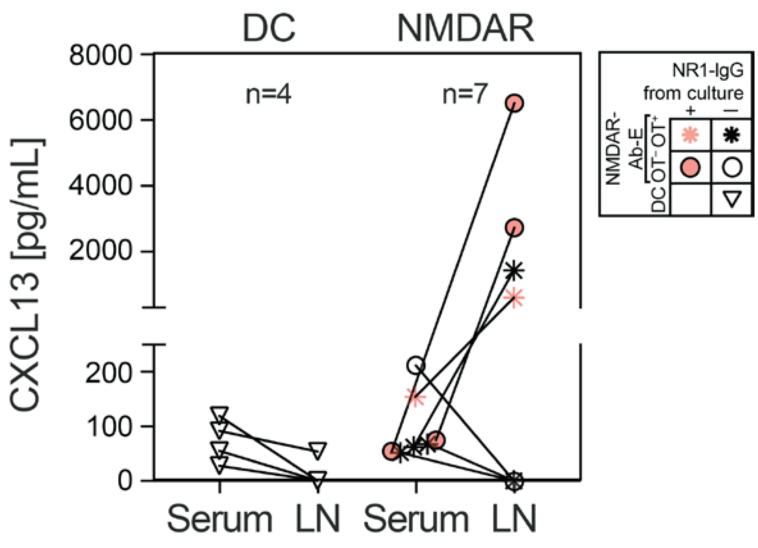

Supplementary Figure 10. CXCL13 levels in paired serum and lymph node aspirate wash samples. Disease control samples are represented by inverted triangles and patients with NMDAR-antibody encephalitis are represented by a circle if there was no associated ovarian teratoma, or a star if there was an associated teratoma. Cervical lymph node sample that produced NR1-IgG in culture are colored pink.

Abbreviations used: CXCL13 = C-X-C motif ligand 13 , DC = disease control, Ig = immunoglobulin, LN = lymph node, NMDAR or NMDAR-Ab-E = N-methyl-D-aspartate receptor-antibody encephalitis, NR1 = N-methyl-d-aspartate receptor subunit 1, OT = ovarian teratoma

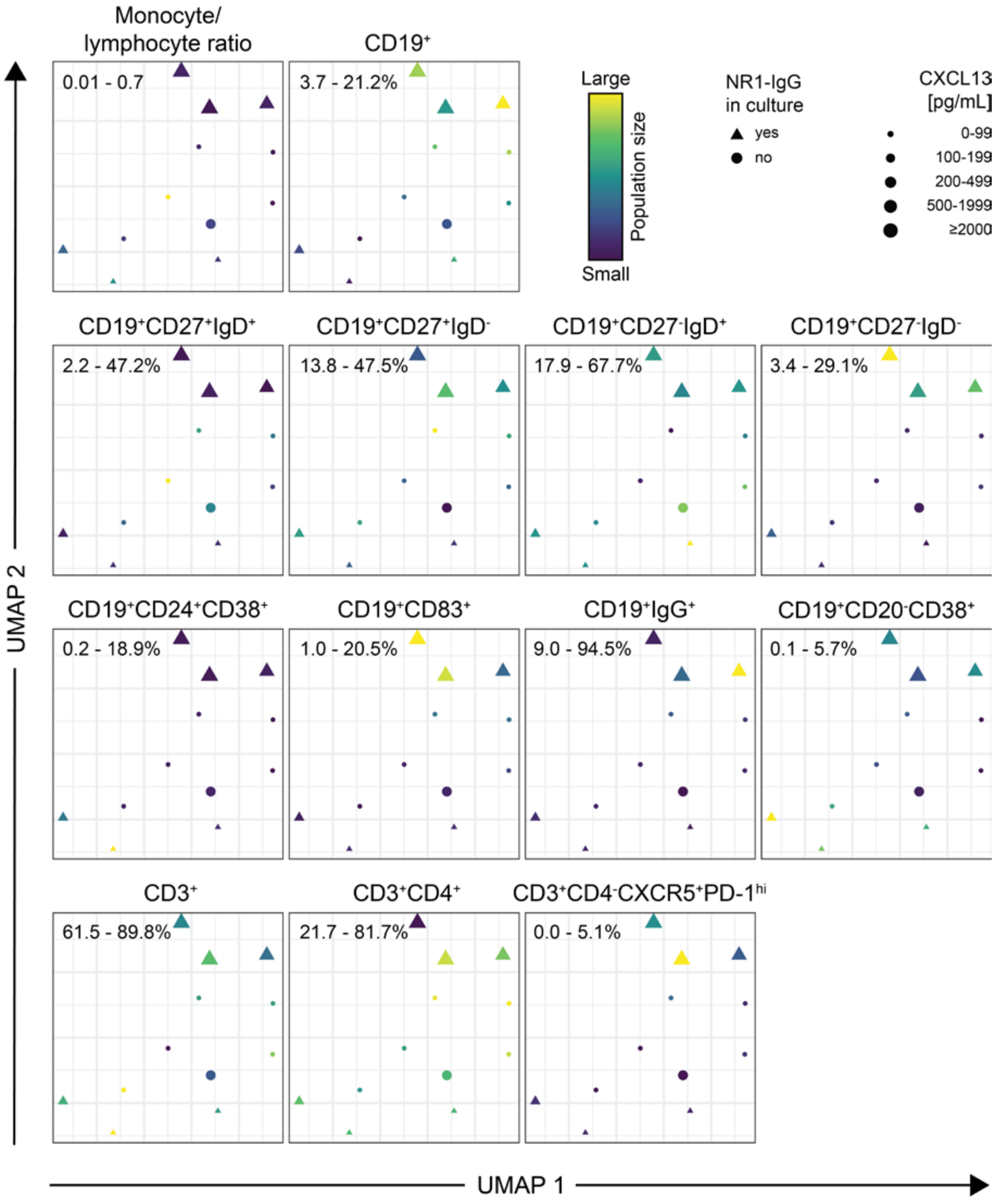

Supplementary Figure 11. The distribution of individual population sizes over UMAP field with relation to Figure 4E. The twelve populations and the monocyte/lymphocyte ratio used to construct the UMAP are displayed individually over the UMAP field. Each dot represents one sample. Colors denote large (yellow) to small (blue) relative percentages of each leucocyte population. $CD3^+$ and $CD19^+$ percentages are based on confirmed lymphocyte singlet population size. Samples that produced IgG autoantibodies against NR1 are represented as triangles whereas those that did not are circles. The size of each point is scaled by the level of CXCL13 detected in paired serum or lymph node aspirate wash sample.

Abbreviations used: CXCL13 = C-X-C motif ligand 13, CXCR5 = C-X-C chemokine receptor type 5, Ig = immunoglobulin, NR1 = N-methyl-d-aspartate receptor subunit 1, PD-1 = programmed death 1, UMAP = uniform manifold approximation and projection for dimension reduction
